# Solvent‐Induced Covalent Bond Softening Boosts Battery Voltage

**DOI:** 10.1002/anie.9887882

**Published:** 2026-04-14

**Authors:** Yanyan Wang, Zhijie Wang, Mengzi Geng, Chunzhen Yang, Guangchao Li, Jean‐Marie Tarascon, Biao Zhang

**Affiliations:** ^1^ Department of Applied Physics The Hong Kong Polytechnic University Hong Kong China; ^2^ School of Materials Sun Yat‐sen University Shenzhen China; ^3^ Department of Applied Biology and Chemical Technology The Hong Kong Polytechnic University Hong Kong China; ^4^ Chimie du Solide‐Energie UMR 8260 Collège de France Paris France

**Keywords:** bond elongation, charge transfer, covalent bond weakening, Solvent/redox center interactions

## Abstract

Increasing cell voltage is a key strategy for enhancing the energy density of lithium batteries. Previously, this was mainly achieved by adjusting the redox potentials of transition‐metal‐based cathode materials through inductive effects that altered the covalency of metal—oxygen bonds. Here, we present a novel strategy for increasing battery voltage that consists of acting on the redox potential of the electrochemically active electrode through charge transfer with the electrolyte. To demonstrate this new concept, we used CF*_x_*‐type electrodes, which are found in commercial primary batteries, and successfully achieved an impressive increase in redox potential of over 250 mV. This was done by increasing the ionicity of the C─F bond via a lactam‐based electrolyte with high electron‐donating capability. This finding, which was extended to other electrodes, namely I_2_, was rationalized through an array of analytical techniques and computational methods. Contrary to common belief, we clearly demonstrate that the electrolyte itself can significantly impact the bulk redox properties of electrodes, such as voltage. The new proposed inductive effect, driven by interactions between the solvent and the redox center, opens up new avenues of research in chemical bond regulation. It would also be highly valuable in energy‐related systems, including electrocatalyst and beyond.

## Graphical abstract

The strong attractive interaction between the nucleophilic center of the solvent and the electrophilic region of the covalently bonded cathode fragment triggers charge transfer. This results in the electron population in the antibonding orbital of the covalent bond, leading to bond weakening and elongation, and consequently increasing the cathode potential.

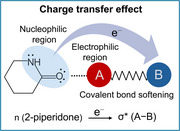

## 1 Introduction

**FIGURE 1:**
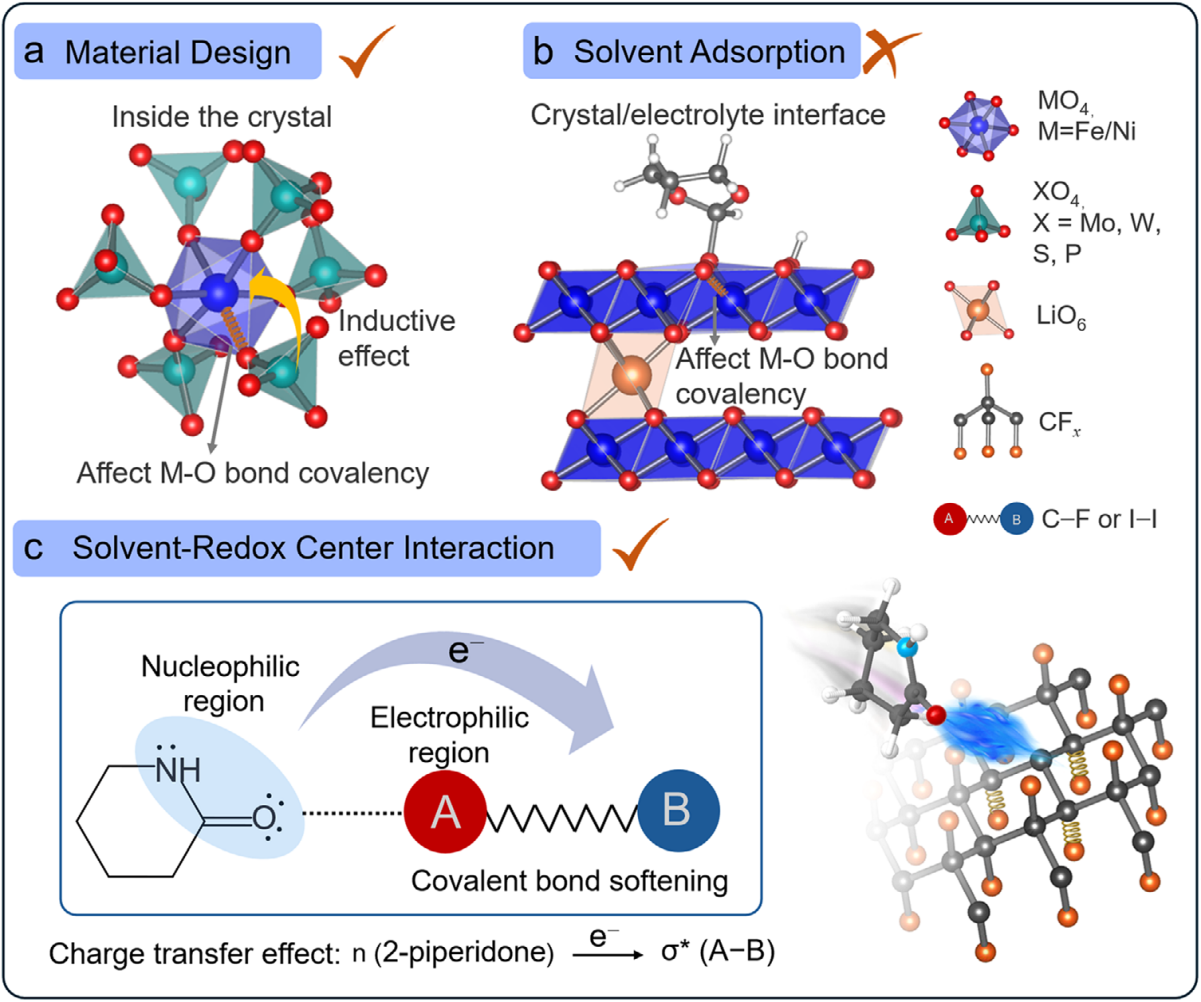
Illustrations of strategies for electrode potential modulation. (a) Material design approach: In Fe_2_(XO_4_)_3_ cathodes, the redox potential correlates with M─O bond covalency, which can be tuned via polyanion (XO_4_) selection. (b) Solvent adsorption at crystal/electrolyte interface: Local changes in M─O bond covalency at the interface do not alter the bulk redox potential. (c) Solvent‐induced covalent bond softening strategy: Charge transfer effect occurs from the lone pair electrons of the amide group to the antibonding orbital of the covalent bond of the cathode, which weakens C─F or I─I bonds.

Energy density is a critical metric in assessing battery performance, depending on the specific capacity of the electrode and the potential difference between the cathode and anode [1, 2]. Although considerable research has been devoted to increasing capacities through novel material design and nanostructuring approaches, comparatively less attention has been given to modulating cell voltage. This disparity stems from the greater complexity involved in potential engineering, which requires precise control over the electronic structure of redox centers and their chemical environments. Successful modulation of the potential has been demonstrated by adjusting the electron configuration of the redox center in transition metal oxide cathodes, where the redox potential of the M^n+^/M^(n+1)+^ couple is significantly correlated with its coordination environment [3, 4]. Consequently, the cathode potential can be modified by adjusting the bonding characteristics between transition metal cations and their surrounding anions or anionic ligands through the inductive effect [5, 6]. This is clearly demonstrated in NASICON‐type cathodes [7], where the equilibrium potential of M_2_(XO_4_)_3_ (where M represents a transition metal, and X is selected from Mo, W, S, P) can be increased from 3.0 to 3.6 V when the polyanion changes from Fe_2_(MoO_4_)_3_ to Fe_2_(SO_4_)_3_. The counterions exert an inductive effect through the M–O–X linkages, influencing the strength of the M–O covalency [8] (Figure 1a). While the formation of such an inductive effect is primarily limited to the design of the cathode structure, the M─O bond can be affected by the interaction with solvent when the electrode is immersed in the electrolyte. We have previously demonstrated that the degree of covalency of the Ni─O bond in LiNi_0.8_Co_0.1_Mn_0.1_O_2_ could be reduced following adsorption of the solvents [9] onto the surface as schematically shown in Figure 1b. However, this does not result in noticeable changes in the redox potential due to i) relatively weak influence on the M─O bond and ii) the weak impact on long‐range electronic structure since only the cathode surface comes into contact with the electrolyte. Inspired by the established correlation between cathode potential and covalent bond characteristics, we speculate that cathode potential could be regulated if the redox centers have sufficient contact with solvents. The extent of the potential shift would depend on the properties of the solvent in the electrolytes. Figure 1c illustrates how the strong attractive interaction between the nucleophilic center of the solvent molecule (the lone pair electrons on the amide group) and the electrophilic region of the covalently bonded fragment of the cathode triggers charge transfer. This results in the electron population in the antibonding orbital of the covalent bond, leading to bond weakening and elongation, and consequently increasing the cathode potential.

To verify the hypothesis, we adopted the carbon fluoride (CF*_x_*), a covalent‐bonded cathode material featuring high energy density and having significant practical value, as a model system. The CF*_x_* is typically synthesized through fluorination of carbon precursors, which retains the layered structure of the original carbon material to facilitate solvent infiltration [10, 11] (Figure S1 and Note S1). The electrochemical reaction [12] proceeds as follows: $CF_{x}+xLi\to C+xLiF$. The nature of the C─F bonds in CF*_x_* is highly distinctive, which directly correlates with the electrochemical performance. The theoretical capacity is proportional to the value of *x* (0.865 Ah g^−1^ when *x* = 1) while the discharge voltage usually decreases as *x* increases. The covalent C─F bond, which dominates in the CF*_x_* with high fluorine content, yields the lowest operating potential compared to the (semi)‐ionic C─F bonds [13]. However, ionic C─F bonds are extremely difficult to synthesize and typically form only at low fluorine‐to‐carbon ratios (*x* ≤ 0.05) [10]. There is a strong need for approaches beyond material design to resolve this dilemma. In recent years, efforts to elevate the discharge voltage of CF*_x_* have predominantly targeted kinetic limitations. A moderately Lewis‐acidic Sn(OTf)_2_ additive promotes LiF dissolution and its conversion into more conductive SnF_2_, thereby suppressing interfacial polarization, particularly at high rates [14]. Similarly, incorporation of a fluorinated ether cosolvent improves wettability and reduces charge‐transfer resistance, leading to lower polarization and elevated discharge plateaus [15]. Electrolytes containing 1,3‐dimethyl‐2‐imidazolidinone have been reported to decrease the C─F bond dissociation barrier and regulate LiF formation and deposition, thus reducing overpotential and increasing the practical discharge voltage [16]. Huang et al. further introduced isoxazole, a solvent with pronounced π‐hole character, to construct π‐hole⋯F supramolecular interactions that alleviate kinetic barriers and enable high‐rate Li/CF_x_ performance [17]. Importantly, these strategies enhance the observed voltage primarily through kinetic regulation, by mitigating polarization, rather than by modifying the intrinsic thermodynamics of the conversion reaction. Herein, from a thermodynamic perspective, we propose a solvent‐induced covalent bond softening strategy that converts the majority of covalent C─F bond into semi‐ionic bond for improving the discharge voltage while maintaining a high *x* value to obtain an attractive capacity (Figure 1c). This approach can be applied widely, as demonstrated by extending to iodine (I_2_) cathode, which has a unique electronic structure, characterized by low‐energy antibonding orbitals. This enables I_2_ to function as an effective electron acceptor when interacting with solvent molecules [18], thus facilitating the formation of charge‐transfer complexes and realizing a 330 mV positive shift in the I_2_ redox potential. These results reveal that solvent‐derived inductive effects can effectively manipulate the covalent bond strength of cathode materials to improve the working potential.

## 2 Results and Discussion

The efficacy of the solvent‐induced covalent bond softening strategy depends critically on the judicious selection of solvent molecules. The extent to which the electronic configuration modulation of the redox centers is directly related to the strength of solvent‐redox center interactions. These interactions are fundamentally governed by functional group chemistry. We compare solvents that are widely used in batteries, including carbonate ester, phosphate ester, and carboxylic ester, as well as ether. Among these, amides induce the most significant voltage increase in Li||CF*_x_* (*x* equals to approximately 0.95) battery, outperforming carboxylic ester by over 250 mV (Figure S2). This may be due to the strong polarity of the amide group, which is caused by the delocalization of the nitrogen lone pair into the carbonyl π* orbital, forming a conjugated system. This conjugation induces electron density redistribution from nitrogen to oxygen, creating an electron‐rich center at the carbonyl oxygen [19], which is expected to exert intensive intermolecular interactions with the redox centers and influence their electron configuration. However, linear amides are severely incompatible with Li metal anodes due to their high reactivity. Adjusting the molecular structure of amides through cyclization and increasing the ring size effectively mitigates this issue. Lactams with ring sizes of six/seven members are significantly more stable against Li metal and maintain excellent compatibility even at elevated temperatures (Figure S3). Based on these considerations, we use 2‐piperidone, a six‐membered cyclic lactam, to replace the dimethoxyethane (DME) in the conventional electrolyte (CE) where propylene carbonate (PC) is employed as a co‐solvent at a 1:1 volume ratio. The optimized amide‐containing electrolyte (2‐piperidone and PC in the volume ratio of 1:1 as the solvent) is denoted as AE. To ensure the stability of the electrolyte, we select 1 M lithium difluorophosphate (LiPO_2_F_2_) as the primary salt. Note that the lithium salt has a negligible effect on the voltage of Li||CF*_x_* batteries (Figure S4). LiPO_2_F_2_ was selected primarily for its superior electrochemical and thermal stability, which is essential for reliable operation, particularly at elevated temperatures. Compared with other lithium salts, it minimizes parasitic reactions and enables stable battery performance across a wide temperature range, ensuring accurate evaluation of the intrinsic cathode behavior. ^13^C liquid‐state nuclear magnetic resonance (NMR) spectroscopy was employed to probe the Li^+^ coordination environments in AE and CE. For AE, the significant downfield shift (0.86 ppm) of the 2‐piperidone carbonyl carbon indicates strong Li^+^‐carbonyl oxygen coordination and the dominance of 2‐piperidone in the primary solvation shell (Figure S5). The negligible shift of PC confirms its minor role, consistent with its lower donor number compared to 2‐piperidone (15 vs 30, Figure S6). In contrast, both PC and DME in CE show comparable downfield shifts, demonstrating their co‐participation in the Li^+^ solvation (Figure S7).

**FIGURE 2:**
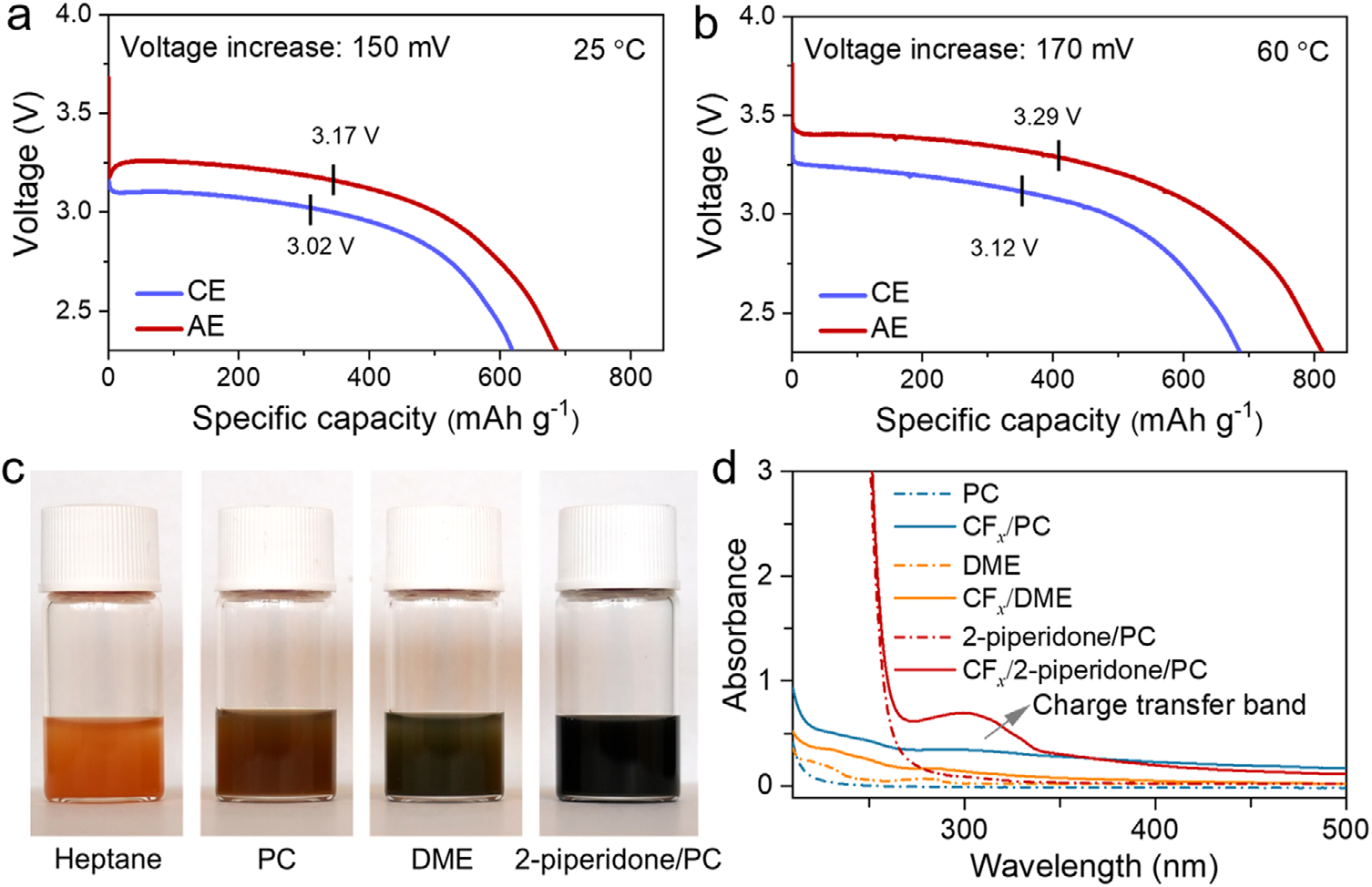
Distinct behavior of the CF*_x_* battery in two electrolytes. The discharge voltage profiles of the Li||CF*_x_* battery using AE and CE as electrolyte, respectively, measured at (a) 25°C and (b) 60°C with a current density of 10 mA g^−1^. The black marks represent the voltage at the midpoint of specific capacity. (c) The solvent‐induced effect causes color changes in CF*_x_*. (d) The electronic absorption spectra of CF*_x_* suspension using PC, DME, and 2‐piperidone/PC as solvents, respectively, and their corresponding pure solvents.

Figure 2a shows the discharge voltage profiles for Li||CF*_x_* batteries employing AE and CE at a current density of 10 mA g^−1^ (∼1/80 C‐rate). At 25°C, the AE system demonstrates a medium discharge voltage of 3.17 V, representing a 150 mV enhancement over the CE system (3.02 V), leading to a 15% increase in energy density (Figure S8). This effect cannot be attributed to ohmic polarization due to the difference in ionic conductivity since the AE has a lower ionic conductivity than the CE (2.4 × 10^−4^ S cm^−1^ vs 3.8 × 10^−4^ S cm^−1^), as further confirmed by the observation of identical discharge voltages in the high‐ionic‐conductivity CE‐LiBF_4_ electrolyte (2.9 × 10^−3^ S cm^−1^) (Figure S9). Due to the extremely low current rate, the physical property of electrolytes has a negligible effect on voltage. Galvanostatic intermittent titration technique (GITT) measurements were performed to determine the equilibrium potentials at identical states of charge. The relaxed voltages (dashed lines) confirm that the increased potential in the AE electrolyte persists under equilibrium conditions, indicating that the enhancement originates from thermodynamic effects rather than differences in kinetic polarization (Figure S10). AE system maintains an advantage in discharge voltage at elevated temperatures, achieving enhancements of 170 mV at 60°C (Figure 2b) and 180 mV at 100°C (Figure S11) over the CE system to improve the energy density (Figure S8). The solvent effect works for the majority of fluorinated sites in the CF*_x_* cathode, as evidenced by the uniform potential elevation throughout the discharge process. Rate capability tests performed under current densities of 10∼1000 mA g^−1^ show that increasing current density leads to reduced capacity and lower discharge voltage due to enhanced polarization (Figure S12).

One might attribute the voltage variations to the reaction pathways in different electrolytes, given that early studies hypothesized the presence of intermediate phases or complexes during discharge [20, 21]. However, contemporary studies employing advanced characterization techniques have consistently identified crystalline LiF and carbon as the discharge products, showing no evidence of Li^+^‐intercalated phases or intermediate species [12]. Tracking the discharge process via ex situ x‐ray diffraction (XRD) (Figure S13) reveals a progressive fading of the CF*_x_* (001) diffraction peak. This is accompanied by a concomitant increase in the carbon (002) and LiF signals. This evolution is indicative of a conversion reaction and is observed in both AE and CE. Time‐of‐flight secondary ion mass spectrometry (TOF‐SIMS) analysis of discharged cathodes from both AE and CE systems further confirms it (Figure S14 and S15), showing mainly LiF and carbon signatures. This suggests that the reaction pathway is the same, involving the direct conversion of CF*_x_* into carbon and LiF. Another possible cause of the voltage variation may arise from Li^+^ activity‐dependent anode electrode potential. While the Nernst equation predicts anode potential dependence on Li^+^ activity and experimental studies demonstrate that the Li/Li^+^ redox potential can vary by up to 600 mV across different solvent systems, such modulation affects both electrodes equally in full‐cell configurations [22, 23]. Specifically, the anode and the cathode undergo a shift of equivalent magnitude in the same direction, resulting in a net‐zero change in the overall cell voltage. To verify the influence of Li^+^ activity on battery voltage, ferrocene (Fc) was used to calibrate the Li/Li^+^ redox potential. Measurements revealed a negative shift of 100 mV (Figure S16) of the Li/Li^+^ potential in diluted AE electrolyte (−3.74 V vs. Fc/Fc^+^) compared to standard AE (−3.64 V vs. Fc/Fc^+^). However, this variation does not result in an improved output voltage in Li||CF*_x_* cells (Figure S17). Identical behavior is observed in CE systems, proving that changes in Li^+^ activity cannot explain the operational voltage changes of Li||CF*_x_* batteries. It is worth noting that the phenomenon is not limited to a particular carbon fluoride. Similar voltage enhancement has been observed for the sample prepared from a different precursor, fluorinated graphite (*x*≈1.03 in CF*_x_*) (Figure S18). As this sample does not possess rich nanopores, it suggests a minor effect of porosity on the solvent‐induced bond softening. Instead, the intimate contact between solvent and electrode is enabled by solvent infiltration into the layered structure, as discussed in Note S1.

We speculate that the voltage increase achieved in the system arises from strong interactions between the solvent and CF*_x_*, a theory that is partly supported by the distinct solvent‐dependent coloration of CF*_x_*: pale orange in non‐polar heptane, light brown in PC, dark grey in DME, and deep black in 2‐piperidone/PC solutions (Figure 2c). This chromatic change demonstrates variations in the electronic structure of CF*_x_* upon interaction with solvents, which is further corroborated by the corresponding electronic absorption spectrum. When CF*_x_* is dispersed in a mixed solvent of 2‐piperidone and PC, a distinct new absorption peak emerges at around 300 nm. This feature is barely perceptible in pure PC or DME solvent systems (Figure 2d). The newly observed absorption band likely originates from the strong interfacial interaction between the interface of CF*_x_* and 2‐piperidone, inducing a significant charge transfer effect between the two components. Concentration‐dependent UV—vis measurements of the CF_x_/2‐piperidone/PC system (10∼30 mg ml^−1^) show a progressive increase in the charge‐transfer band intensity with increasing CF*_x_* content, supporting its assignment to 2‐piperidone/CF_x_ interactions (Figure S19.). Moreover, comparison with structurally distinct solvents reveals that the characteristic charge transfer band appears only in the presence of 2‐piperidone (Figure S20). The appearance of this characteristic absorption confirms the formation of charge transfer complexes at the CF*_x_*/2‐piperidone interface, which is closely related to the change of C─−F bond energy. Thermodynamic analysis (Note S2) shows that the weakened C−F bonds in Li||CF*_x_* batteries result in higher discharge potentials through reduced bond energy.

**FIGURE 3:**
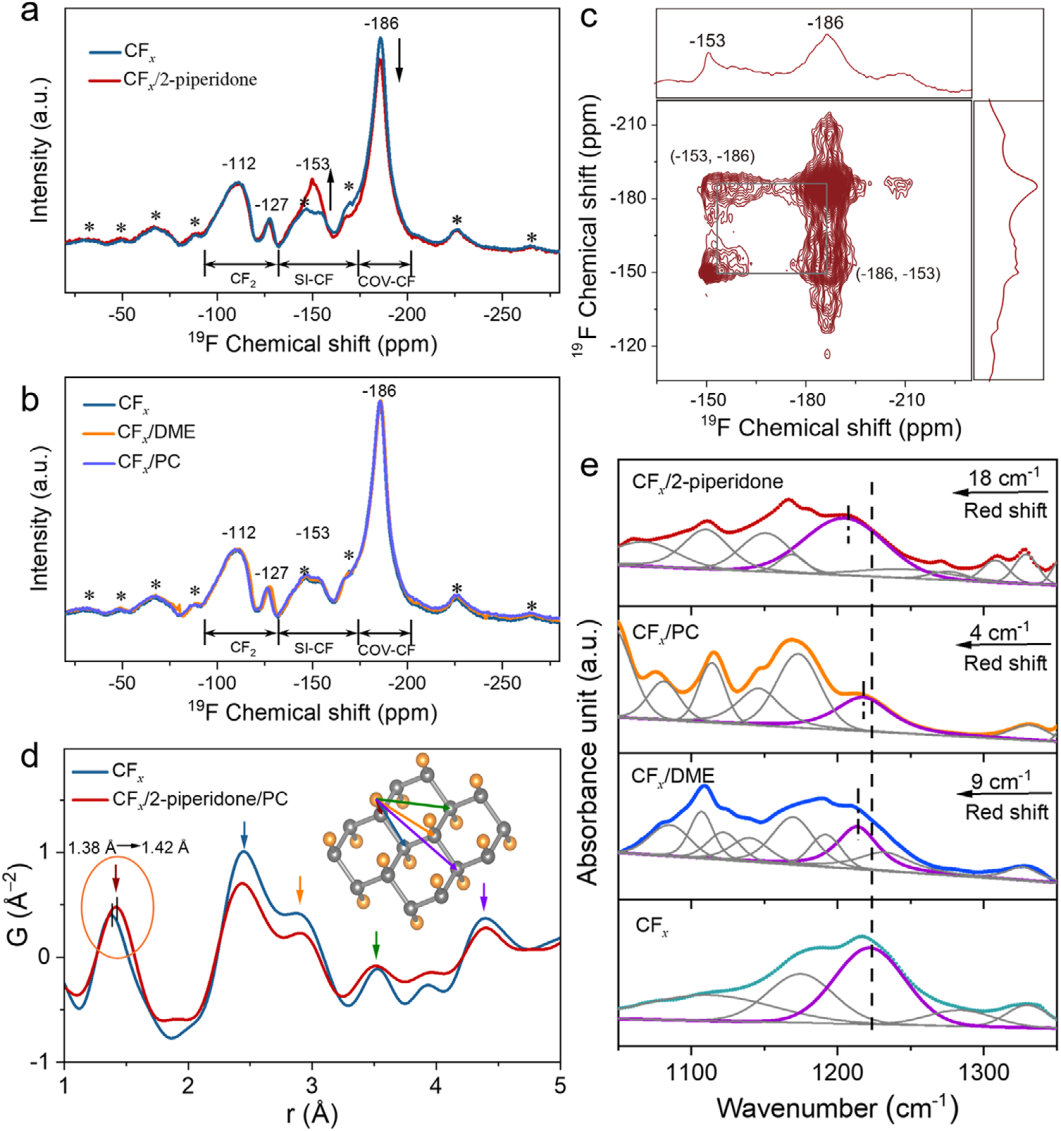
Probing C─F bond. ^19^F solid‐state NMR spectra of CF*_x_* with and without adsorption of (a) 2‐piperidone, (b) DME and PC, spinning at 18 kHz. The spinning sidebands are marked with *. (c) Two‐dimensional ^19^F—^19^F homonuclear exchange MAS solid‐state NMR spectra of CF*_x_* adsorbed with 2‐piperidone, with a recoupling period of *τ *= 200 ms. (d) High‐energy x‐ray total scattering derived PDF for pristine CF*_x_* and CF*_x_* absorbed with 2‐piperidone/PC. (e) The FTIR spectra of pristine CF*_x_* powders and CF*_x_* absorbed with 2‐piperidone, PC, and DME, respectively.

The variation of the electronic structure of CF*_x_* materials induced by solvent molecules can be detected by examining fluorine chemical shift changes via solid‐state ^19^F NMR spectroscopy. Pristine material displays four fluorine signatures consisting of a covalent C─F bond (COV‐CF) at −186 ppm, a semi‐ionic bond (SI‐CF) at −153 ppm, and two CF_2_ edge groups at −127 and −112 ppm [13]. Following 2‐piperidone adsorption, the intensity of the covalent C−F signal at −186 ppm diminishes while the semi‐ionic peak at −153 ppm intensifies; the CF_2_ environments remain unaffected (Figure 3a). The selective signal evolution indicates a preference for 2‐piperidone to interact with monofluorinated carbon sites. This reduces the covalency of the C─F bond, facilitating the conversion to a semi‐ionic character, which is consistent with an increase in discharge potential. As 2‐piperidone loading increases, the covalent C−F signal at −186 ppm progressively attenuates. This demonstrates that higher concentrations of 2‐piperidone promote more extensive conversion of covalent C−F bonds to semi‐ionic character (Figure S21). In contrast, CF*_x_* powders adsorbed with DME or PC show no detectable changes in their ^19^F NMR spectra compared to pristine CF*_x_* (Figure 3b). The absence of spectral shifts confirms minimal interactions between the solvent and CF*_x_*, proving that PC and DME lack the capability to soften covalent C─F bonds. Two‐dimensional ^19^F—^19^F magic angle spinning (MAS) exchange solid‐state NMR spectroscopy reveals the fluorine environments in 2‐piperidone adsorbed CF*_x_* powders in detail. At 20 ms recoupling time (Figure S22), cross‐peaks emerge between −153 and −186 ppm, suggesting that covalently and semi‐ionic bonded F atoms are in close spatial proximity (<5 Å) and interact via dipolar coupling [24, 25]. Extending the recoupling time to 200 ms (Figure 3c) intensifies these cross‐peaks, demonstrating the time‐dependent phenomenon of chemical exchange, which is induced by the mobility and diffusion of adsorbed 2‐piperidone molecules. Specifically, when a 2‐piperidone molecule approaches a particular C−F pair, it can induce a transition from a covalent to a semi‐ionic bond. Conversely, the C─F bond reverts to its covalent state when the 2‐piperidone molecule moves away. The dynamic adsorption and desorption of 2‐piperidone on the CF*_x_* causes the ^19^F nuclei to switch between the two chemical environments, which proves that C─F bond softening is induced by 2‐piperidone. The above NMR analysis shows how solvents affect the distribution of electron density around F atoms, supporting the electronic absorption spectrum and electrochemical conclusions on the changes in electron configuration.

To further interrogate the structural evolution of CF*_x_* in more detail upon solvent absorption, an analysis of the atomic pair distribution function (PDF) was conducted using synchrotron x‐ray total scattering. This technique provides the probability of distribution of interatomic distances, where the positions of the peaks correspond to separations between atomic pairs and the widths of the peaks reflect structural disorder. As shown in Figure 3d, pristine CF*_x_* exhibits a broad peak profile, indicating significant structural disorder. The C–F nearest‐neighbor distance appears at 1.38 Å, corresponding to the average C─F bond length. Upon absorption of the 2‐piperidone/PC, the C─F bond elongates to 1.42 Å, which directly demonstrates the softening of the covalent bond induced by the solvent. This trend is also observed in the fluorinated graphite with and without solvent interaction (Figure S23).

We then use Fourier‐transform infrared (FTIR) spectroscopy to characterize the solvent‐dependent C─F bond changes in CF*_x_*. Pristine CF*_x_* powder exhibits a characteristic broad absorption band at 1100—1300 cm^−1^ corresponding to C─F stretching vibrations. This band undergoes a pronounced 18 wavenumber cm^−1^ redshift upon interaction with 2‐piperidone, indicating C─F bond weakening. In contrast, control systems with PC and DME show shifts of merely 4 and 9 wavenumbers cm^−1^, respectively (Figures 3e and S24). The solvent‐induced covalent bond softening strategy developed here overcomes a key limitation of conventional materials synthesis methods. While direct preparation of F‐rich CF*_x_* with high semi‐ionic C─F bond content remains infeasible, our method successfully converts most of the covalent C─F bonds to semi‐ionic configurations, thereby boosting the voltage, even when the fluorine‐to‐carbon ratio in the CF*_x_* material is as high as 0.95.

**FIGURE 4:**
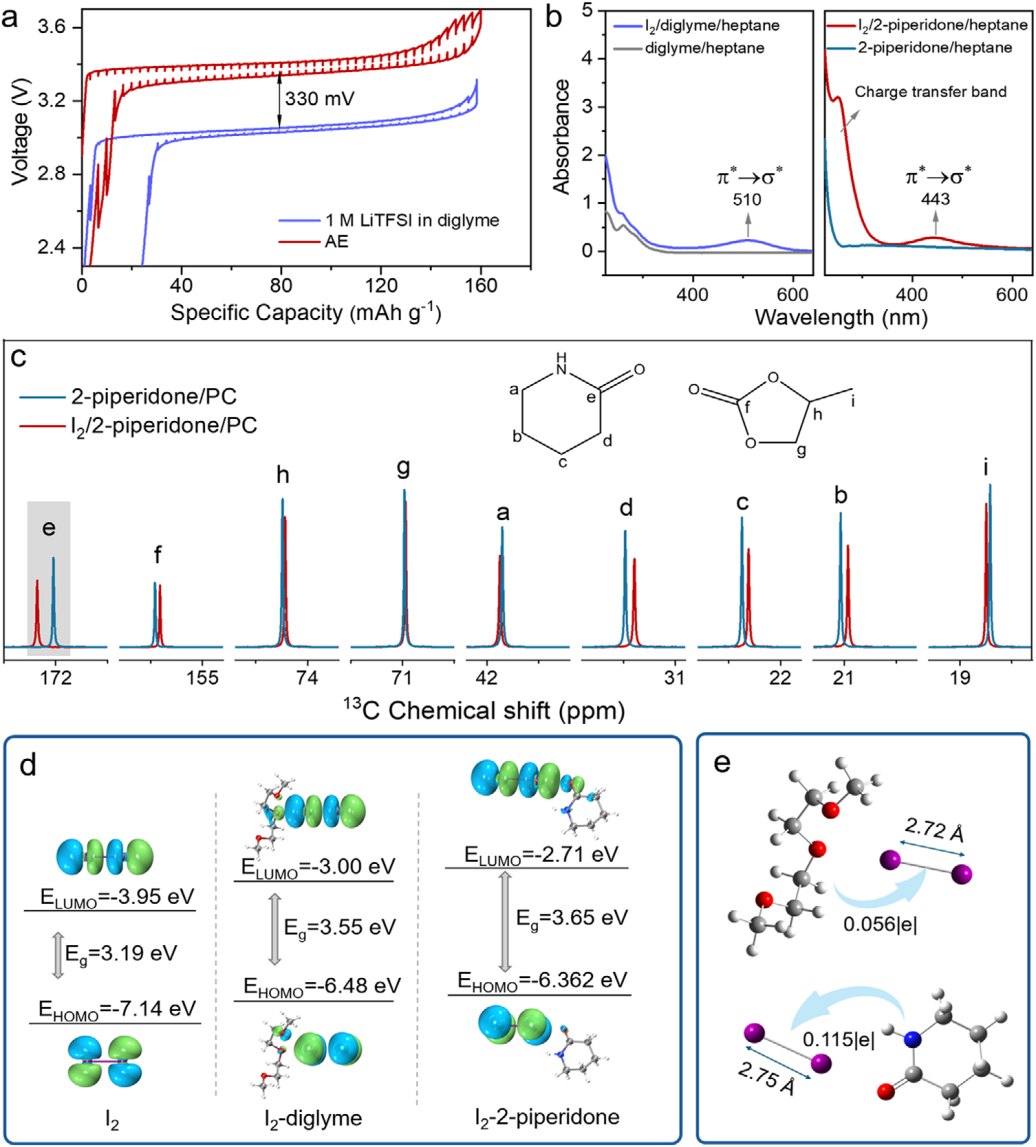
Solvent effects on I_2_ cathode. (a) GITT test of Li||I_2_ batteries using the two electrolytes. The solid lines are the experimental GITT curves while the dashed lines represent the equilibrium potential after each relaxation process. (b) Electronic absorption spectra of I_2_ dissolved in 2‐piperidone/heptane and diglyme/heptane solutions. (c) The ^13^C NMR spectra of 2‐piperidone/PC mixture with and without I_2_. (d) The distributions and energy level of the LUMO and HOMO orbitals of I_2_, I_2_‐diglyme complex and I_2_‐2‐piperidone complex. (e) Charge transfer between the solvent and the I_2_ molecule.

The above observations manifest the effectiveness of the solvent‐induced covalent bond softening strategy in raising the cathode potential. This effect is based on the premise of intimate contact between a high‐polarity solvent and a redox center, making it most powerful in the cathode with an open‐frame structure (such as CF*_x_*) or an unsheltered redox center. This is demonstrated by its extension to rechargeable Li||I_2_ battery systems, in which the cathode consists of covalently bonded I_2_, exhibiting a particularly strong susceptibility to solvent interactions and electronic modulation. Galvanostatic intermittent titration technique (GITT) measurement (Figure 4a) reveals that a Li||I_2_ battery employing classical 1 M LiTFSI/diglyme electrolyte delivers a reversible equilibrium potential of approximately 3.0 V, corresponding to the I^−^/I^0^ redox transition, whereas a battery utilizing AE (identical to the one used in the CF*_x_* system) achieves a 330‐mV enhancement. Notably, such a gain in the voltage does not compromise capacity or cycling stability (Figure S25). In addition, the AE electrolyte enables the two‐electron I^−^/I^+^ redox reaction in Li||I_2_ batteries. The I^0^/I^+^ redox transition is observed at 3.7∼4.0 V (Figure S26), representing an increase of approximately 400 mV compared with previously reported values [26].

In order to investigate the solvent‐dependent potential phenomenon in Li||I_2_ batteries, the FTIR and ^13^C NMR spectroscopy were employed to examine the molecular interactions between I_2_ and solvent species. The FTIR spectra revealed distinct red shifts in characteristic vibrational modes of 2‐piperidone upon I_2_ dissolution. Notably, this occurred in the C═O stretch at 1650 cm^−1^ and N─H stretches at 3191 and 3297 cm^−1^, identifying the amide group as the primary binding site for I_2_ (Figure S27). However, vibrational modes of diglyme barely change upon the addition of I_2_ (Figure S28). This conclusion is further corroborated by ^13^C NMR analysis (Figures 4c and S29), which shows that the carbonyl carbon of 2‐piperidone shifts downfield by 0.16 ppm upon I_2_ addition, whereas PC shows negligible changes. By contrast, I_2_ only interacts weakly with the terminal methyl group of diglyme, as evidenced by minimal chemical shift changes (Figure S30). Solvent‐I_2_ interactions greatly influence the electron configuration of I_2_ molecules, as demonstrated by the distinct changes in UV—vis spectroscopy (Figure 4b). In non‐polar heptane (dipole moment: 0 D), I_2_ exhibits a π*→σ* transition at 520 nm [27] (Figure S31), while this transition undergoes blue shifts to 510 nm with addition of diglyme (dipole moment: 1.92 D) and a further blueshift to 443 nm with the addition of 2‐piperidone (dipole moment: 4.27 D). This indicates strong solvent‐induced polarization of I_2_ molecules [28] (Figure 4b). Furthermore, the I_2_/2‐piperidone/heptane solution exhibits a distinct charge‐transfer band. This is the result of electron donation from the lone pair of the 2‐piperidone to the I−I σ* orbital, followed by the formation of a charge‐transfer complex. Similarly, the charge‐transfer band intensity increases with increasing I_2_ concentration, following the same trend observed for CF*_x_* (Figure S32). However, this phenomenon is less perceptible in the I_2_/diglyme/heptane solution. The amide group in 2‐piperidone demonstrates particularly strong electronic coupling with I_2_, a phenomenon rooted in its electron‐rich character. The electron‐rich site participates in robust electrostatic interactions with I_2_, whose high polarizability enables significant electron cloud distortion.

Density functional theory (DFT) calculations provide additional insight into solvent‐dependent modifications of electronic structure in I_2_ systems. The computational results demonstrate that the formation of charge transfer complexes between I_2_ and polar solvents increases the HOMO‐LUMO gap. 2‐piperidone induces the most dramatic expansion of the bandgap to 3.65 eV, which correlates with the observed blue shift of the π*→σ* transition in the electronic absorption spectra (Figure 4d). Charge population analysis reveals that the formation of charge transfer complexes between I_2_ and solvents involves electron donation from solvent molecules to the antibonding orbitals of I_2_, with 2‐piperidone showing the greatest amplitude of charge transfer (see Figures S33 and S34). This electron transfer weakens the I─I bond, increasing its length from 2.72 Å in I_2_/diglyme to 2.75 Å in I_2_/2‐piperidone complexes (Figure 4e). These length changes distinctly impact the redox potential, with longer I─I bonds corresponding to higher electrochemical potential. Both theoretical and experimental results demonstrate that 2‐piperidone engages in the strongest intermolecular interactions with I_2_, causing the most significant perturbation to its electron configuration.

The fundamental similarity between Li||CF*_x_* and Li||I_2_ battery systems is their covalent‐bonded feature, where the voltage is associated with bond strength. The developed solvent‐induced bond softening strategy creates pronounced inductive effects that weaken these covalent bonds, thereby enabling improvement of the cathode potential. We mainly focus on the use of 2‐piperidone to convey our message, but it goes without saying that alternative amide‐based molecules could be used, as demonstrated with the other molecules. Of course, work remains to be done to establish the effects of cyclic vs non‐cyclic molecules and to adjust the length of the alkane chain.

Together, these findings highlight the importance of solvent‐derived inductive effect, which has been disregarded until now, in boosting cell voltage and, consequently, energy density. This could offer a transformational change in the way to design better electrode materials, particularly when it comes to overcoming contradictory structural and compositional demands, as seen with CF*_x_*, to secure high voltage without compromising capacity. Furthermore, the family of covalent‐bonded cathodes is diverse, and it is worth revisiting electrodes besides CF*_x_* and I_2_. One example is organic cathodes, where reversible bond cleavage occurs, offering an opportunity to adjust the redox potential by softening the bonds with suitable solvents as demonstrated in our initial explorations (Figure S35 and Note S3). Lastly, from a materials perspective, the regulation of chemical bonds, with substantial efforts predominantly centering on materials design, is critical in many other applications. For example, the surface bonding state of electrocatalysts largely determines their activity. This solid‐liquid interaction‐derived inductive effect provides new opportunities to precisely tune the bond strength at the interface. It is noteworthy that this strategy also has certain limitations. In densely packed crystalline materials, restricted solvent infiltration into the bulk structure confines charge transfer complex formation primarily to surface regions, under which conditions the strategy cannot effectively operate. Furthermore, successful implementation requires suitable energy level alignment and sufficient electronic coupling between the solvent and the cathode; these structural and electronic requirements collectively define the practical boundaries of the approach.

## 3 Conclusion

We demonstrate that the solvent‐induced covalent bond softening strategy is effective in modulating the cell voltage. By utilizing intensive interactions between the solvent and the redox center, particularly with polar solvents such as 2‐piperidone, the electron configuration in covalent‐bonded cathode materials can be significantly modified, thereby leading to a higher redox potential. NMR and electronic absorption spectroscopy analyses confirm that 2‐piperidone induces charge transfer interactions, converting the covalent C─F bonds in CF*_x_* into higher‐potential semi‐ionic configurations and weakening the I─I bonds in I_2_. Li||CF*_x_* battery and Li||I_2_ battery exhibit 150 and 330 mV improvement in potential, respectively. Importantly, this approach circumvents the challenges of direct material synthesis by enabling in situ bond softening through electrolyte design. As an additional advantage, the developed electrolyte containing a polar solvent is its exceptional thermal stability, which enables stable battery operation at elevated temperatures. These findings establish solvent‐mediated strategies for increasing battery voltage and offer a complementary pathway to enhancing energy density through material structural design in conjunction with solvents that possess adjusted electron‐donating capability.

## Author Contributions

**Yanyan Wang**: conceptualization, methodology, investigation and data collection, data analysis, writing – original draft, writing – review & editing. **Zhijie Wang**: investigation and data collection, data analysis. **Mengzi Geng**: data analysis. **Chunzhen Yang**: PDF and SAXS analysis. **Guangchao Li**: solid state NMR analysis. **Jean‐Marie Tarascon**: data analysis, writing – review & editing. **Biao Zhang**: conceptualization, methodology, data analysis, writing – original draft, writing – review & editing.

## Conflicts of Interest

The authors declare no conflicts of interest.

## Supporting information




**Supporting File 1**: anie72183‐sup‐0001‐SuppMat.docx.

## Data Availability

The data that support the findings of this study are available from the corresponding author upon reasonable request.
