# Supplementary material for: Solvent‐Induced Covalent Bond Softening Boosts Battery Voltage
Source: Angew Chem Int Ed Engl. 2026 Apr 14;65(22):e9887882. doi: 10.1002/anie.9887882 (PMC13206179; doi:10.1002/anie.9887882)
Supplement: Supplementary file 1 — Supporting File 1: anie72183‐sup‐0001‐SuppMat.docx. [file ANIE-65-e9887882-s001.docx]

Supporting Information for

**Solvent-Induced Covalent Bond Softening Boosts Battery Voltage**

Yanyan Wang,^[1]^ Zhijie Wang,^[1]^ Mengzi Geng,^[1]^ Chunzhen Yang,^[2]^ Guangchao Li,*^[3]^ Jean-Marie Tarascon,^[4]^ and Biao Zhang*^[1]^

**This PDF file includes:**

Methods.

Supplementary note 1-3.

Supplementary Figures. S1 to S35.

**Methods**

Chemicals:

The CF*_x_* with a fluorine content of around 60% was purchased from Xiamen Zhongke Ceffone Technology Co., Ltd. Lithium difluorophosphate (LiPO_2_F_2_, 99.9%), lithium hexafluorophosphate (LiPF_6_, 99.9%), propylene carbonate (PC, 99.99%), dimethoxyethane (DME, 99.9%), ethylene carbonate (EC) and diethyl carbonate (DEC) were purchased from DodoChem. Lithium nitrite (LiNO_3_, 99.99%) and diglyme (99.9%), iodine (I_2_, 99.9%), and 3,4,9,10-perylenetetracarboxylic dianhydride (PTCDA, 98%) were purchased from Sigma-Aldrich. 2-pyrrolidone (99%), caprolactam (99%), and lithium iodide (LiI, 99.9%) were purchased from Macklin. 2-piperidone (98%) was purchased from Leyan.

Electrochemical testing:

To prepare the CF*_x_* electrode, a slurry composed of active materials, Super P and polyvinylidene difluoride (PVDF) at a mass ratio of 8:1:1 was cast onto Al-foil and followed with drying at 80 ºC for 12 h. Then, the electrode was cut into small disks with a diameter of 1.2 cm. The mass loading of the CF*_x_* cathode is around 2 mg cm^−2^. The PTCDA electrode was prepared using the same method. To prepare the LiI electrode, an aqueous slurry composed of LiI, Super P, and carboxymethyl cellulose (CMC) at a mass ratio of 4:4:2 was cast onto carbon cloth and followed by drying at 60 ºC for 2 h. The mass loading of the LiI cathode is around 0.6 mg cm^−2^. The GITT test was performed on a Solartron Analytical 1400 Cell Test System. The Li||I_2_ batteries and the Li||PTCDA batteries were charged and discharged at 0.1 C, while the relaxation period was terminated either after 4 hours or by limiting dV/dt < 0.1 mV s^−1^. For room-temperature measurements, cells were assembled in CR2032-type coin-cell configuration using a Celgard 2325 membrane as the separator. For elevated-temperature experiments, thermally resistant Swagelok-type cells were employed together with glass fiber separators. Cells containing 2-piperidone/PC-based electrolytes were assembled with glass fiber separators to address wetting issues. In addition, 1 wt% lithium nitrate (LiNO_3_) was added to the electrolytes to stabilize the lithium metal anode during elevated-temperature measurements.

Characterization:

^13^C liquid-state NMR spectroscopy measurements were performed on [a Bruker AVANCE III 400 MHz](https://errd.dicp.ac.cn/info/1050/1538.htm) spectrometer, and the samples were prepared with coaxial inserts in NMR tubes to avoid the influence of deuterated reagents. All ^19^F solid-state NMR spectroscopy measurements were performed on a JEOL ECZ500R 500 MHz spectrometer using a 3.2mm HFX probe. ^19^F chemical shifts were referenced to the trichlorofluoromethane (CFCl_3_) at 0 ppm. Prior to testing, CF*_x_* powders were dehydrated and uniformly mixed with 2-piperidone through thermal vapor adsorption under vacuum at 150 ºC, and the mass ratio of CF*_x_*/2-piperidone is 1:0.02. The one-dimensional ^19^F MAS solid-state NMR spectra were acquired using a 3.2 mm probe at 18 kHz spinning rate. Since the melting point of 2-piperidone is around 40 ºC, to prevent liquefaction and vaporization of 2-piperidone during measurements, samples with higher 2-piperidone loading were acquired at a reduced spinning rate of 10 kHz. The two-dimensional ^19^F-^19^F homonuclear exchange spectra were recorded on a 3.2 mm probe with a MAS speed of 14 kHz.

UV-visible absorption spectroscopy was carried out with a SHIMADZU UV-2550 using a quartz cell with a 1 mm path length. CF*_x_* suspensions (10 mg mL^−1^) were prepared by dispersing CF*_x_* powder in solvents under magnetic stirring for 12 h. The concentration of the I_2_/heptane solution is 10 mg mL^−1^. I_2_/2-piperidone/heptane and I_2_/diglyme/heptane solutions were prepared by adding 20 µl of 2-piperidone or diglyme into 1 ml I_2_/heptane solution (1mg mL^−1^). PTCDA suspensions (2 mg mL^−1^) were prepared by dispersing PTCDA powder in solvents under magnetic stirring for 12 h.

Total scattering data were acquired using two-dimensional detectors (DT X-Panel 4343a FQI-CSV-XH a-Si two-dimensional image plates) at the BL12SW beamline of the Shanghai Synchrotron Radiation Facility (SSRF) with a beam energy of 97.29 keV. The measurements encompassed both Bragg diffraction and diffuse scattering components. Sample materials were encapsulated in polyimide capillaries with a diameter of 1 mm and sealed with epoxy resin. Two-dimensional images were converted into one-dimensional Q-space versus intensity plots by using the Dioptas program with a CeO2 calibration standard. PDF patterns, G(r), were subsequently extracted using the PDFgetX3^[1]^ software, covering a Q-range of up to 22.5 Å^−1^.

DFT computation: The geometry of these molecules and anions were all optimized under the framework of density of functional theory (DFT) with B3LYP functional^[2-4]^ and def2-TZVP basis set^[5]^. All these DFT calculations were performed using Gaussian 16 program. The visualization of the frontier molecular orbitals were rendered using Visual Molecular Dynamic program (VMD)^[6]^.

**Supplementary Note 1**

CF*_x_* maintains the layered structure of the hard carbon precursor yet exhibits a markedly expanded interlayer spacing due to covalent bonding with fluorine. TEM images reveal its amorphous nature (**Figure S1a**), and the X-ray diffraction (XRD) pattern indicates an interlayer distance of approximately 6.6 Å (**Figure S1c**). Small-angle X-ray scattering (SAXS) is used to characterize internal pore structures in particles across a scale of several tens of nanometers to a few angstroms, operating on the principle of electron density difference between the object and its surrounding medium. The shoulder feature at Q ≈ 0.08Å^−1^ in the SAXS profile of CF*_x_* powder (grey line) originates from spatial correlations between internal pores (**Figure S1b**). Upon solvent infiltration, this feature disappears because the solvent fills the pores and reduces the electron density contrast, leading to an overall decrease in scattering intensity (red line). XRD probes structural changes at an even smaller scale, specifically within the crystalline lattice. For solvent-infiltrated CF*_x_* powders, the (001) diffraction peak shifts toward higher angles, while the (100) plane remains unaffected (**Figure S1c**). This shift suggests that changes in the interlayer spacing may result from the infiltration of solvent molecules. The contraction of the interlayer spacing may originate from a screening effect. Solvent infiltration mitigates the repulsive forces induced by the electronegative fluorine atoms and therefore reduces the interlayer distance. As illustrated in **Figure S1d**, both the pores and interlayers of the CF*_x_* are penetrated by solvent molecules, thus ensuring sufficient interactions between the CF*_x_* and solvents.

**Supplementary Note 2**

The correlation between C−F bond characteristics and the electrochemical potential in Li||CF*_x_* batteries can be rationalized through thermodynamic analysis. Given the discharge reaction of Li||CF*_x_* battery, equation (1), the relationship of electromotive force (E) and the Gibbs free energy change (∆G) can be described as equation (2), where T is temperature, ∆S is entropy change, ∆H is reaction enthalpy, and $F'$ is the Faraday constant. ∆H, formulated in equation 3, includes the average bond enthalpy of the C−F bond, ∆H_(C-F)_. Since the discharge products remain consistent across AE and CE systems, ∆H_1_ maintains a constant value. Equation (5) establishes a relationship between C−F bond strength and cell potential, where reduced ∆H_(C-F)_ values correspond to enhanced electrochemical potentials (E). This thermodynamic analysis provides an approximate framework for understanding the correlation between C−F bond strength and battery voltage, as it does not account for irreversible energy losses during the actual electrochemical reaction and the entropy change across different solvents.

$$CF_{x}+xLi\to C+xLiF (1)$$

$$E=\frac{-\Delta G}{xF'}=\frac{T*\Delta S-\Delta H}{xF'} (2)$$

$$\Delta H=\Delta H_{\left( C-F \right)}+ x\Delta H_{\left( Li \right)}-\Delta H_{C}-x\Delta H_{\left( Li-F \right)}=\Delta H_{\left( C-F \right)}+\Delta H_{1} \left( 3 \right)$$

$$\Delta H_{1}=x\Delta H_{\left( Li \right)}-\Delta H_{C}-x\Delta H_{\left( Li-F \right)} (4)$$

$$E=\frac{T*\Delta S-\Delta H_{\left( C-F \right)}-\Delta H_{1}}{xF'} (5)$$

**Supplementary Note 3**

3,4,9,10-Perylenetetracarboxylic dianhydride (PTCDA), an organic cathode material comprising a perylene core with two conjugated anhydride groups, undergoes reversible Li^+^ storage through carbonyl oxygen redox. The electrochemical reaction involves C=O bond cleavage during lithiation and reformation during delithiation (**Figure S35a**). Electronic absorption spectra analysis reveals 2-piperidone forms charge-transfer complexes with PTCDA, evidenced by a distinct absorption band (**Figure S35b**) corresponding to electron donation from the lone pair of solvent into the C=O antibonding orbital (**Figure S35c**). This interaction weakens the carbonyl bond covalency, in contrast to carbonate solvents (EC/DEC). Electrochemical measurements demonstrate the effectiveness of this strategy- while PTCDA delivers a 2.5 V discharge plateau in conventional EC/DEC electrolyte, the voltage increases by 150 mV to 2.65 V in AE, confirming the general applicability of solvent-induced bond softening for potential regulation.


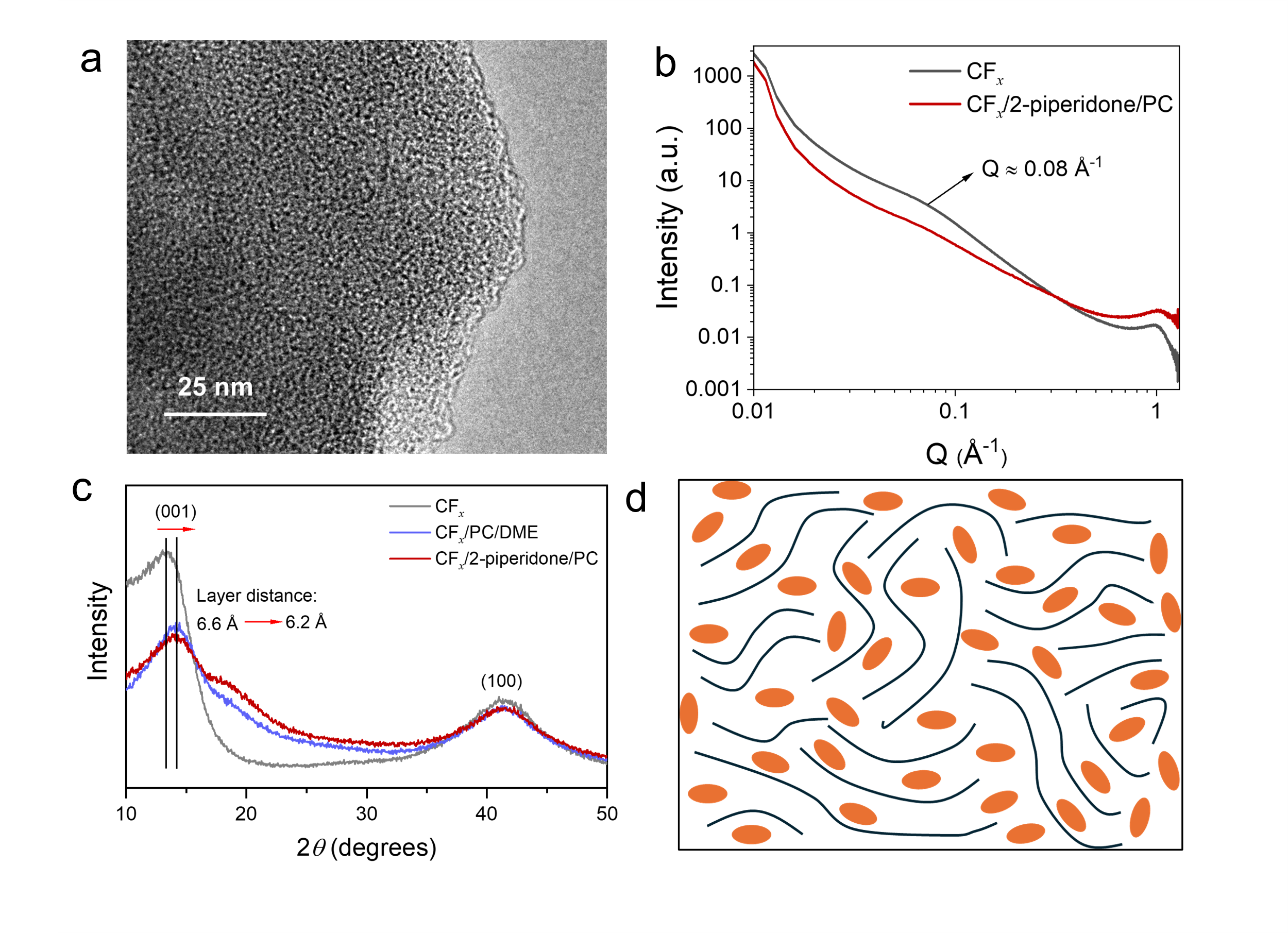


**Figure S1.** Structural information of CF*_x_* and the solvent infiltration phenomenon. (a) TEM image of pristine CF*_x_*; (b) SAXS profile of pristine CF*_x_* and CF*_x_* with absorbed solvent; (c) XRD patterns of pristine CF*_x_* and CF*_x_* with absorbed solvent; (d) Schematic diagram of the distribution of solvent molecules (orange ellipses) in CF*_x_* (black lines). Discussion refers to **Supplementary Note 1**.


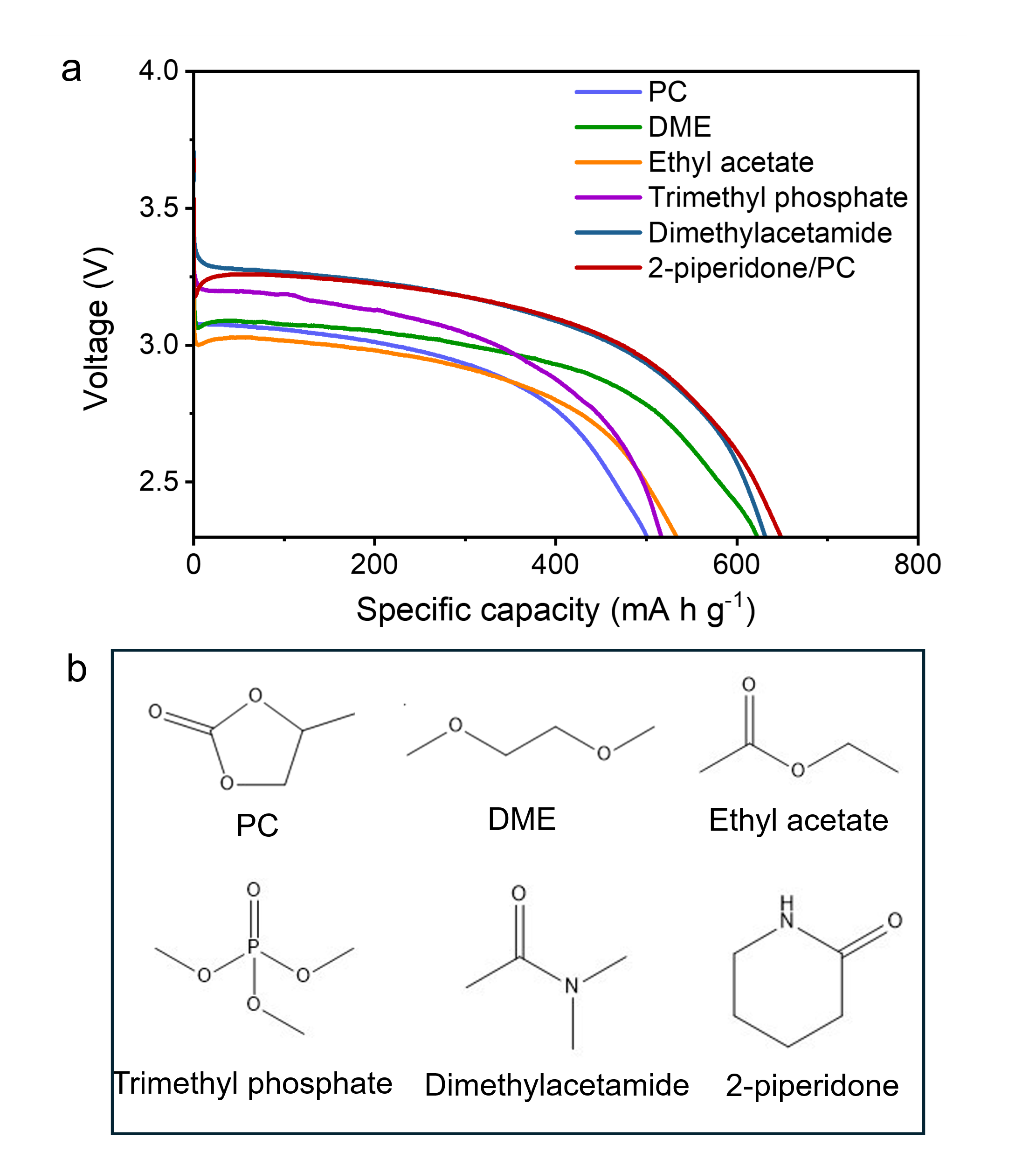


**Figure S2.** (a) The discharge curves of Li||CF*_x_* cell using LiBF_4_ as salt, and PC, DME, ethyl acetate, trimethyl phosphate, dimethylacetamide and 2-piperidone/PC as solvent, respectively. The discharge current density is 20 mA g^−1^. (b) The molecular structure of solvents.

The capacity variation mainly originates from differences in solvent–CF_x_ interfacial compatibility, which affects reaction kinetics and active material utilization, thus influencing the delivered capacity.

**
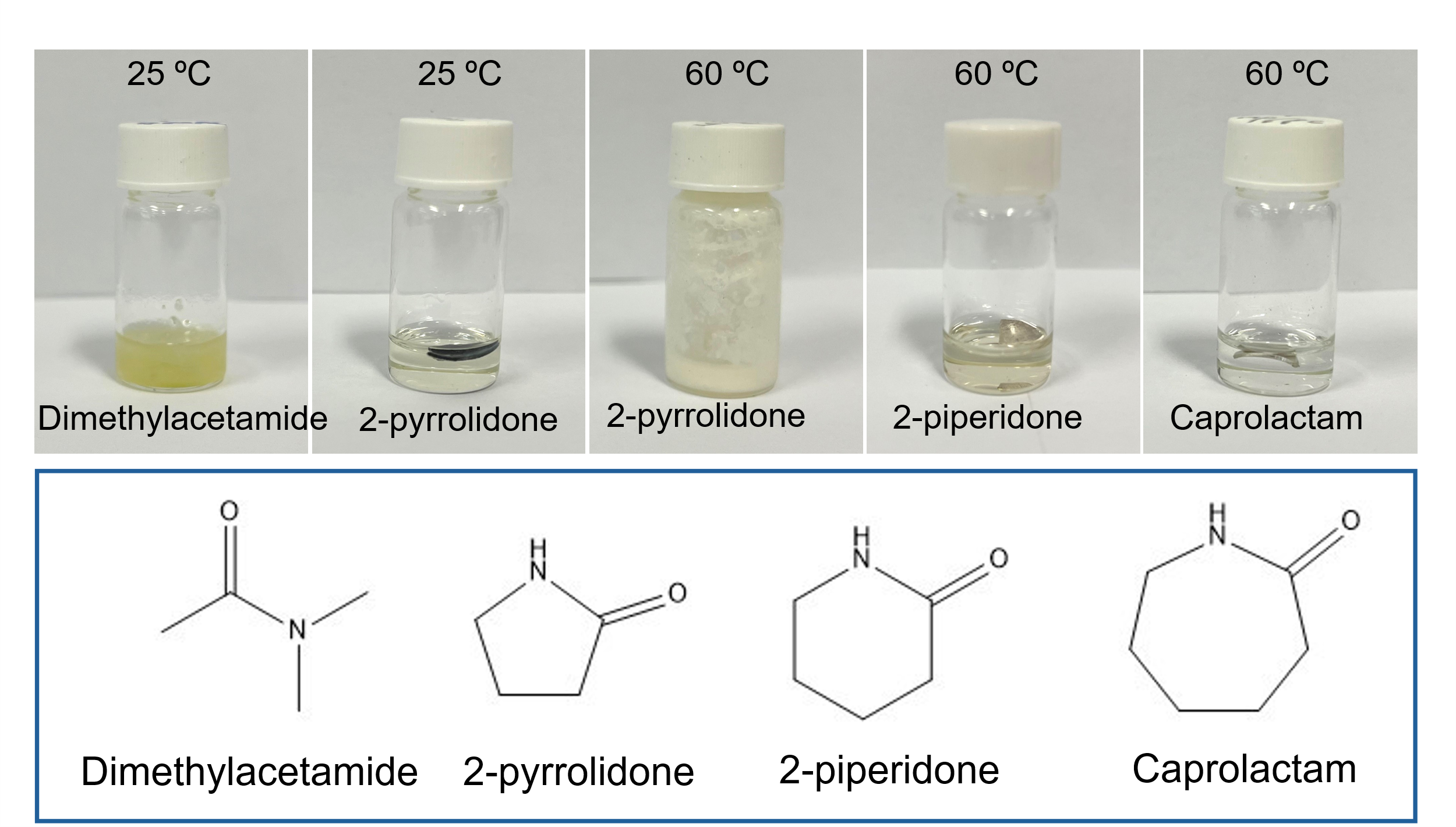
**

**Figure S3.** Compatibility test between Li metal and dimethylacetamide, 2-pyrrolidone, 2-piperidone and caprolactam, respectively.


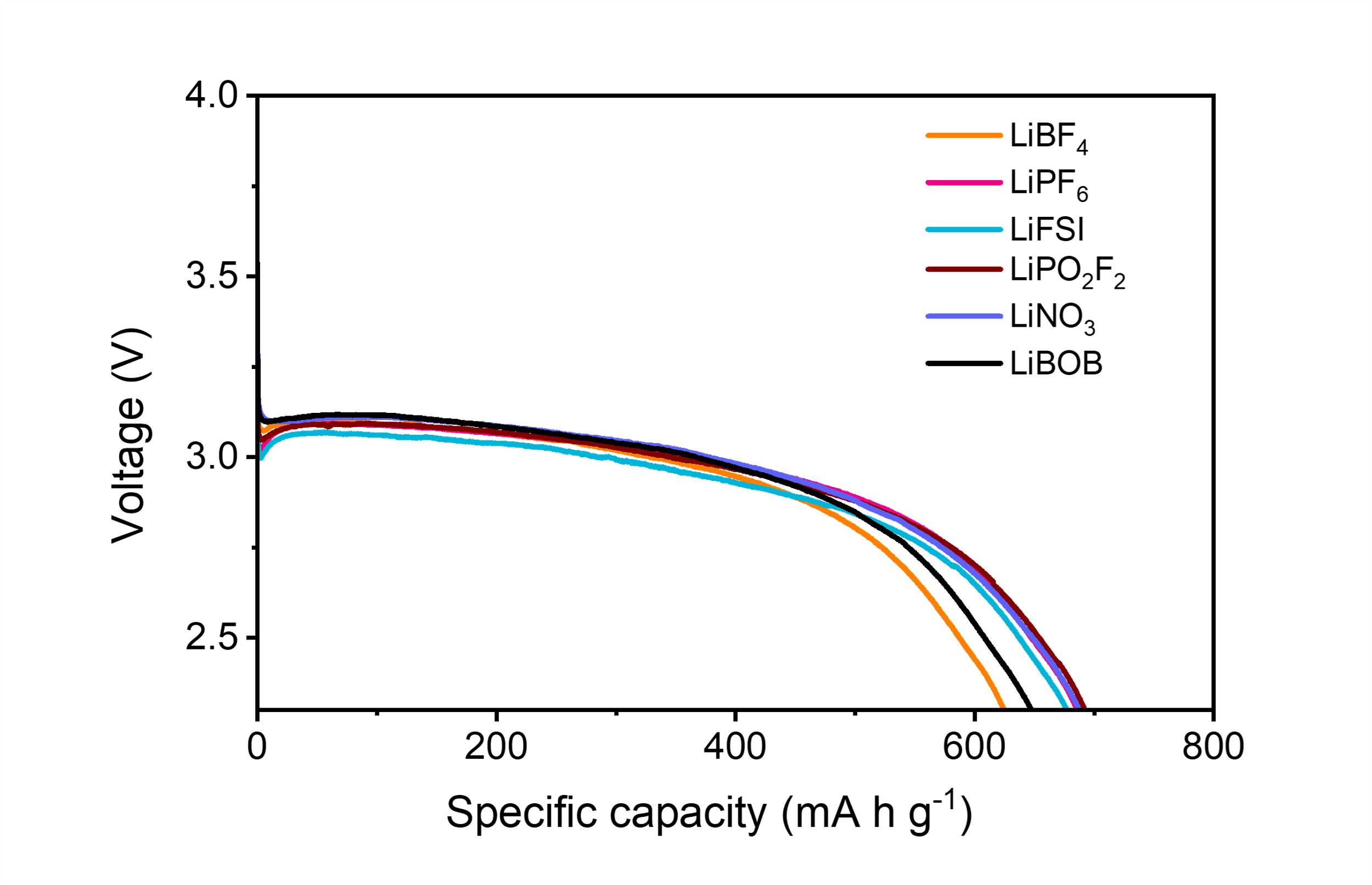


**Figure S4.** The discharge curves of Li||CF*_x_* cell using PC/DME as solvent, and 1 M LiBF_4_, LiPF_6_, lithium bis(fluorosulfonyl)imide (LiFSI), LiPO_2_F_2_, LiNO_3_ and lithium bis(oxalato)borate (LiBOB) as salt, respectively. The discharge current density is 20 mA g^−1^.


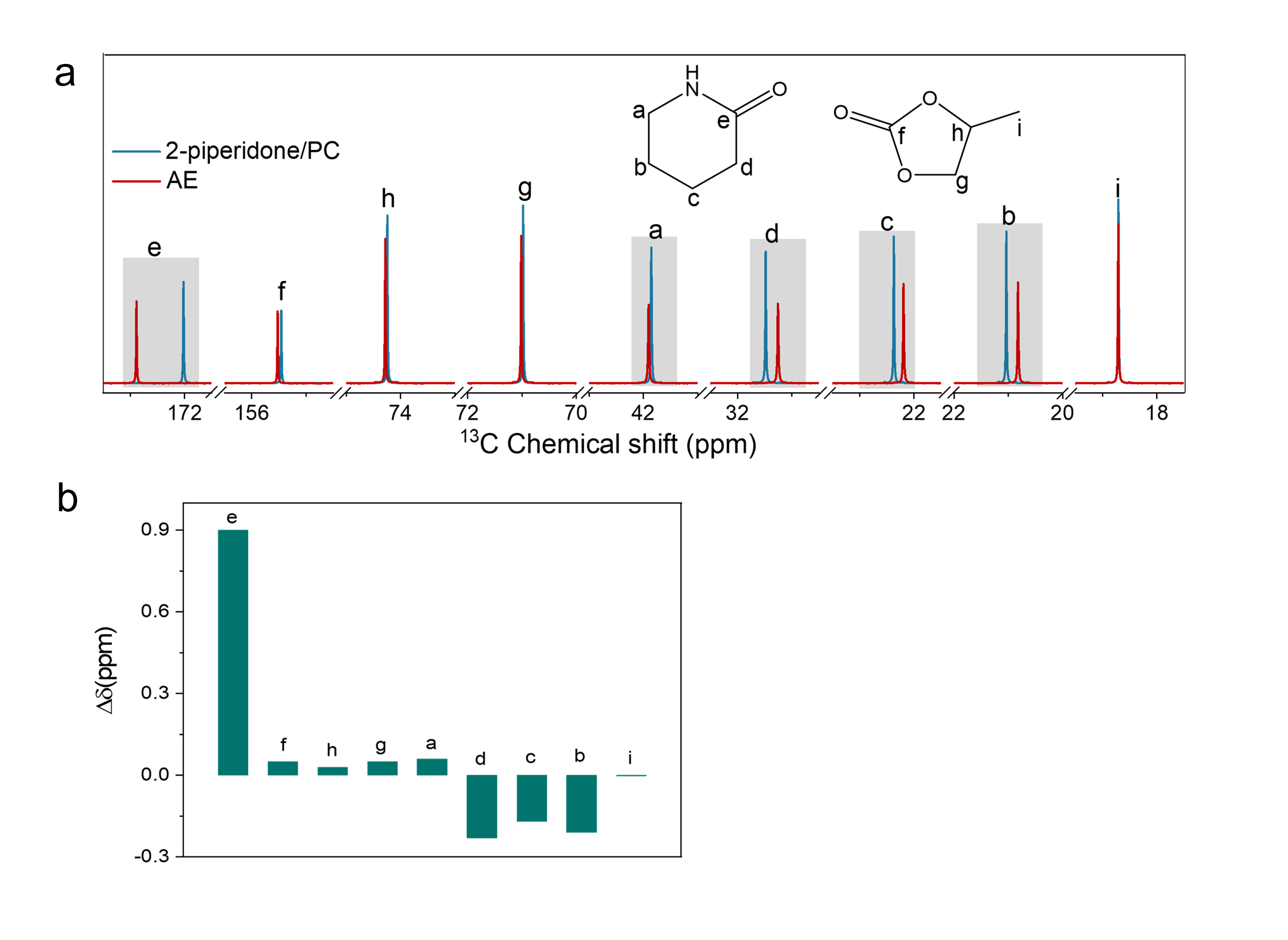


**Figure S5.** (a) The ^13^C NMR spectra of AE and 2-piperidone/PC mixture; (b) chemical shift changes of AE with respect to the 2-piperidone/PC solvent.


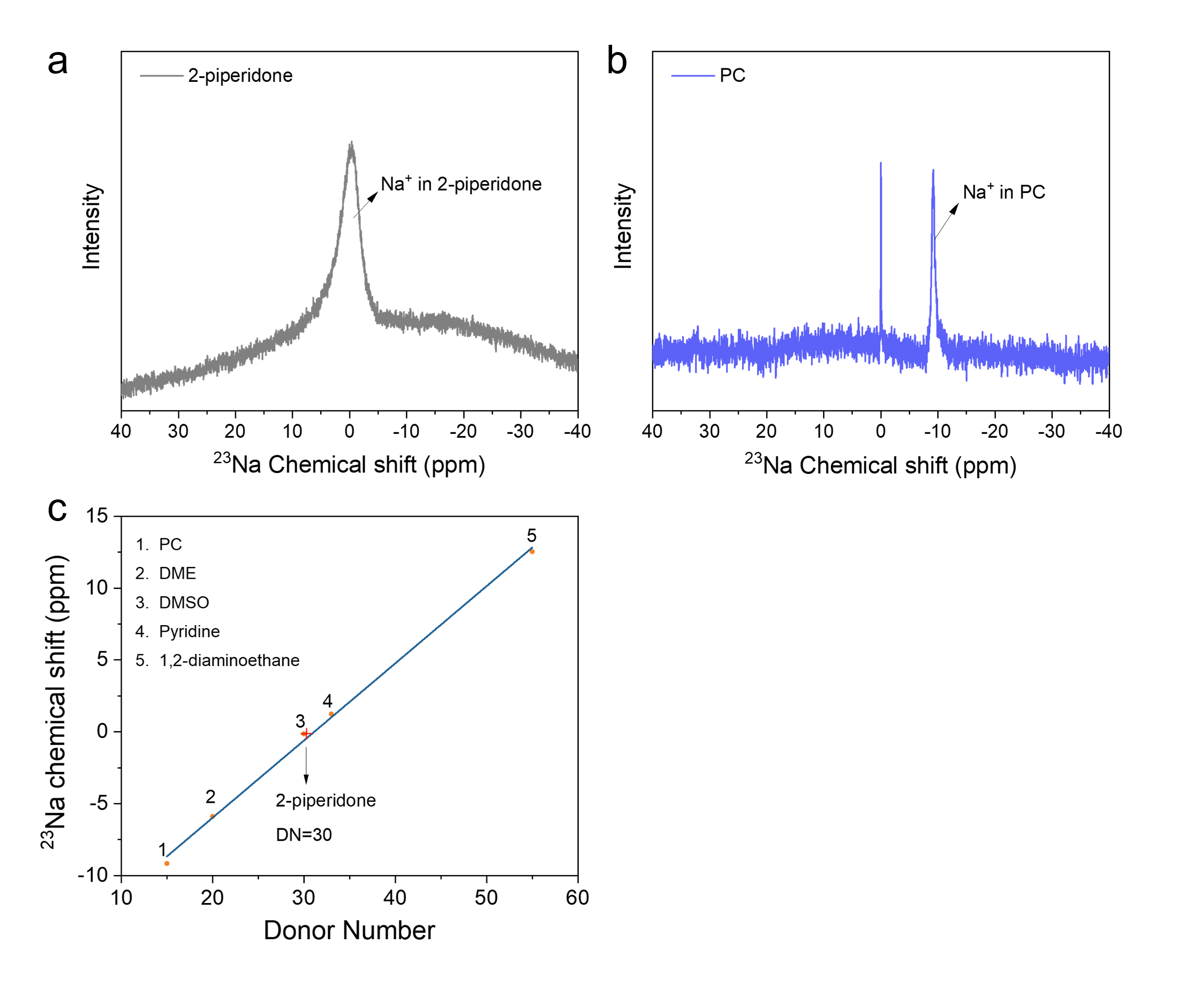


**Figure S6.** The donor number measurement of 2-piperidone via NMR. ^23^Na NMR shift of sodium bis(trifluromethanesulfonyl)imide (NaTFSI) dissolved in (a) 2-piperidone and (b) PC; (c) Plot of ^23^Na NMR shift versus solvent donor number of NaTFSI dissolved in various solvents.

The donor number of 2-piperidone was quantified by exploiting the reported linear correlation between solvent donor number and the ^23^Na chemical shift of NaTFSI in the corresponding solvent^[7]^. Specifically, the ^23^Na chemical shift of NaTFSI/2-piperidone solution (10 mM L^−1^) was measured, and the corresponding donor number was then calculated using the linear formula fitted from solvents with known values.


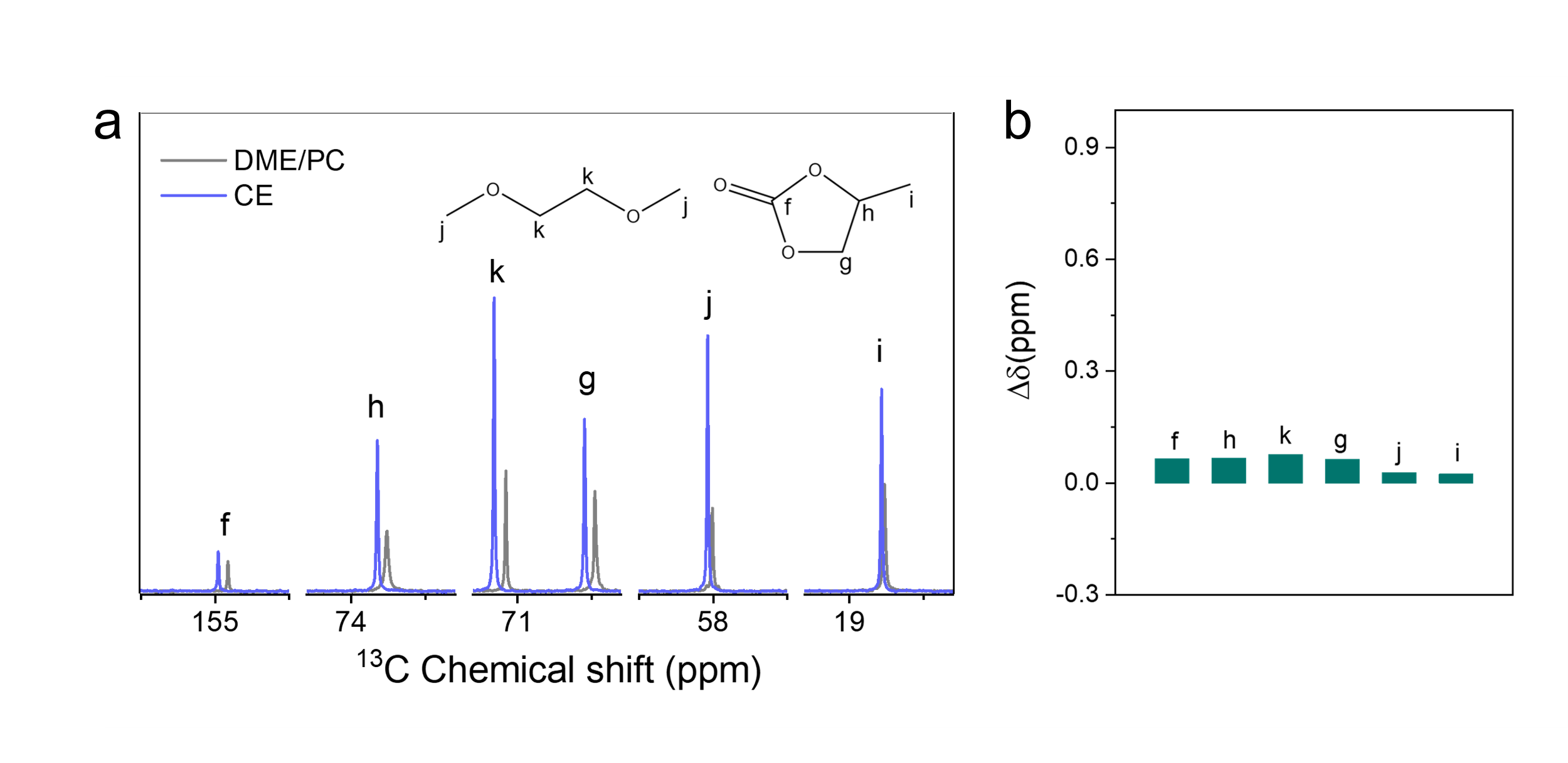


**Figure S7.** (a) The ^13^C NMR spectra of CE and DME/PC mixture; (b) chemical shift changes of CE with respect to the DME/PC solvent.


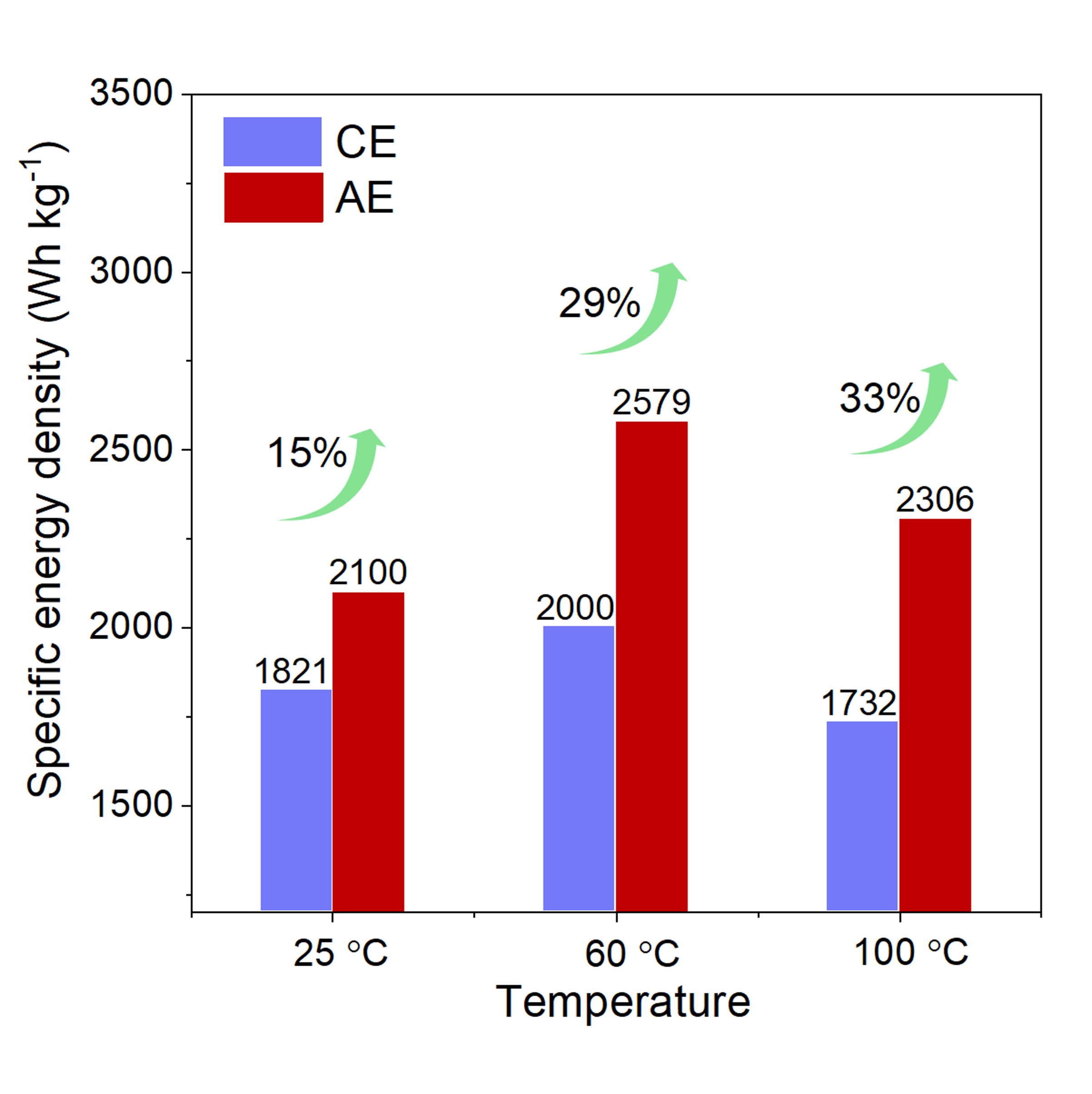


**Figure S8.** The energy density of Li||CF*_x_* battery using AE and CE as electrolyte respectively based on the mass of the cathode. Applied current density is 10 mA cm^−2^.


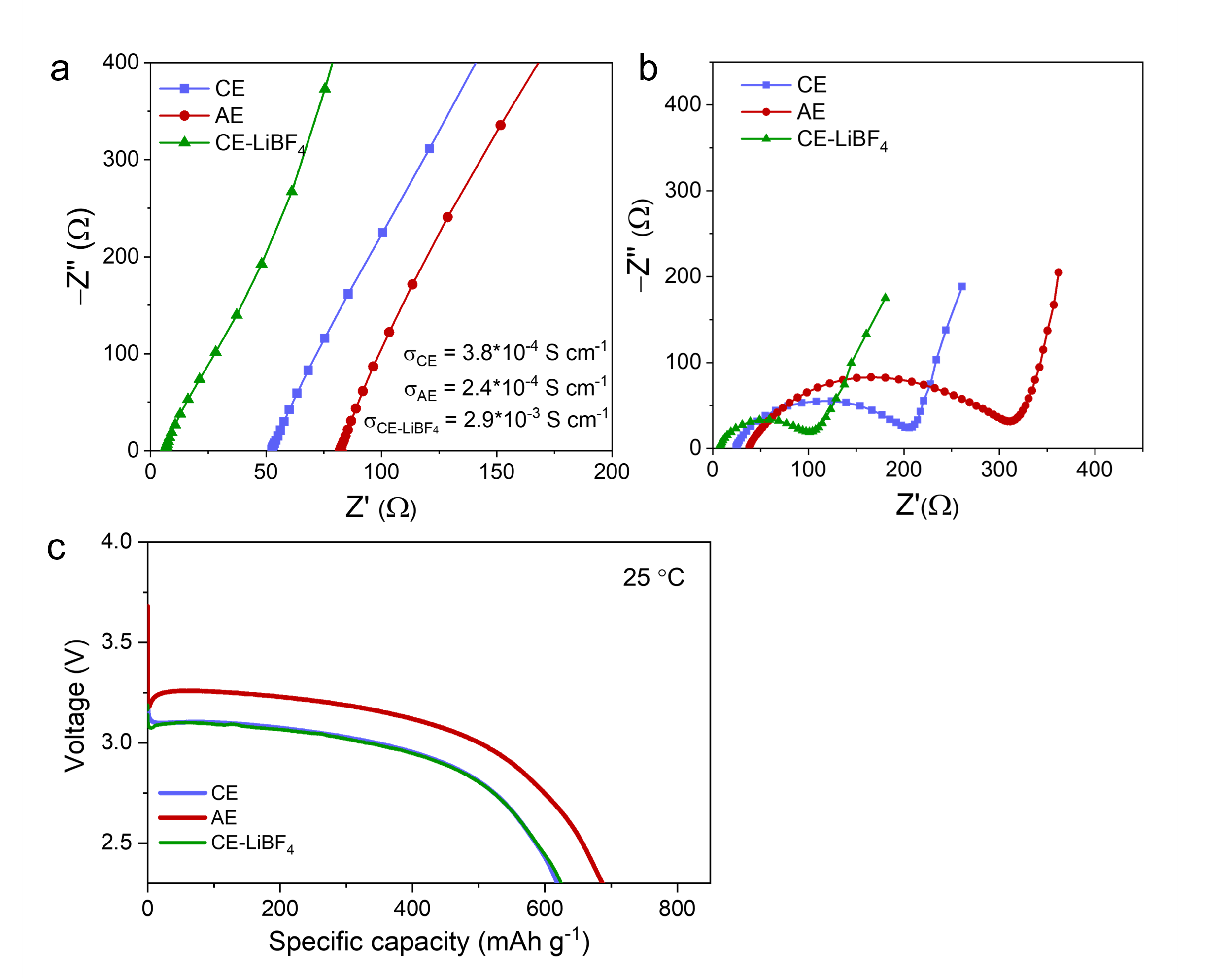


**Figure S9.** (a) The ionic conductivity comparison of CE, AE and 1 M LiBF_4_ dissolved in PC/DME (CE-LiBF_4_); (b) The three-electrode electrochemical impedance spectroscopy (EIS) of CF*_x_* cathode in CE, AE and CE-LiBF_4_, respectively; (c) The discharge voltage comparison of Li||CF*_x_* batteries with CE, AE and CE-LiBF_4_ as electrolyte, respectively.


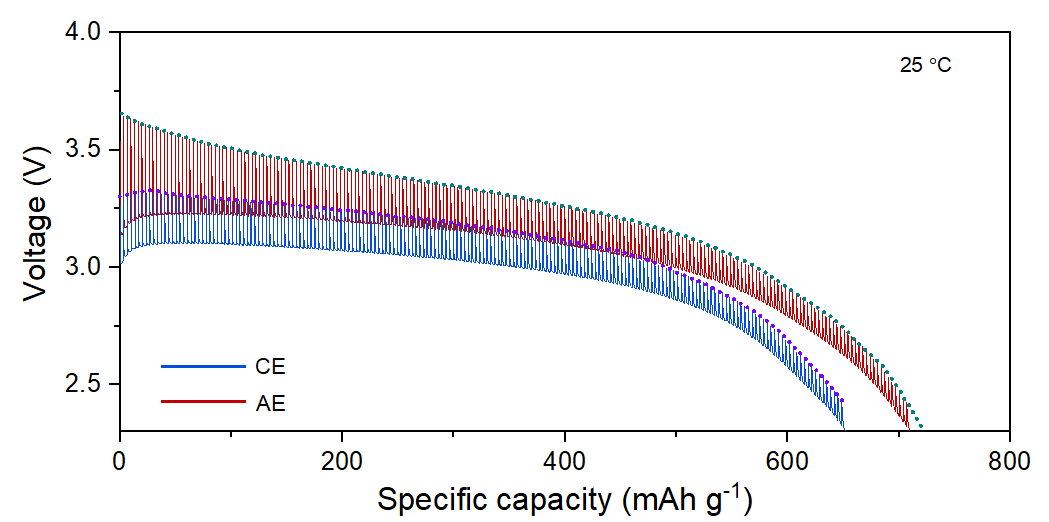


**Figure S10**. GITT test of Li||CF*_x_* batteries using the AE and CE electrolytes.

The difference between the measured discharge potential and the corresponding equilibrium potential (dashed lines) was taken as kinetic polarization. In the Li||CF*_x_* cell with the AE electrolyte, the polarization is approximately 500 mV at the initial stage of discharge. As the reaction proceeds, the polarization decreases to ~180 mV at 50% state of discharge and further declines to ~120 mV near the end of discharge. This gradual reduction is likely associated with improved electronic conductivity of the electrode, arising from the progressive reduction of carbon during discharge. In the CE system, the polarization follows a similar evolution but remains consistently smaller than that in the AE system. This difference is attributed to the higher ionic conductivity of the CE electrolyte compared with the AE electrolyte (3.8×10^−4^ S cm^−1^ vs. 2.4×10^−4^ S cm^−1^).


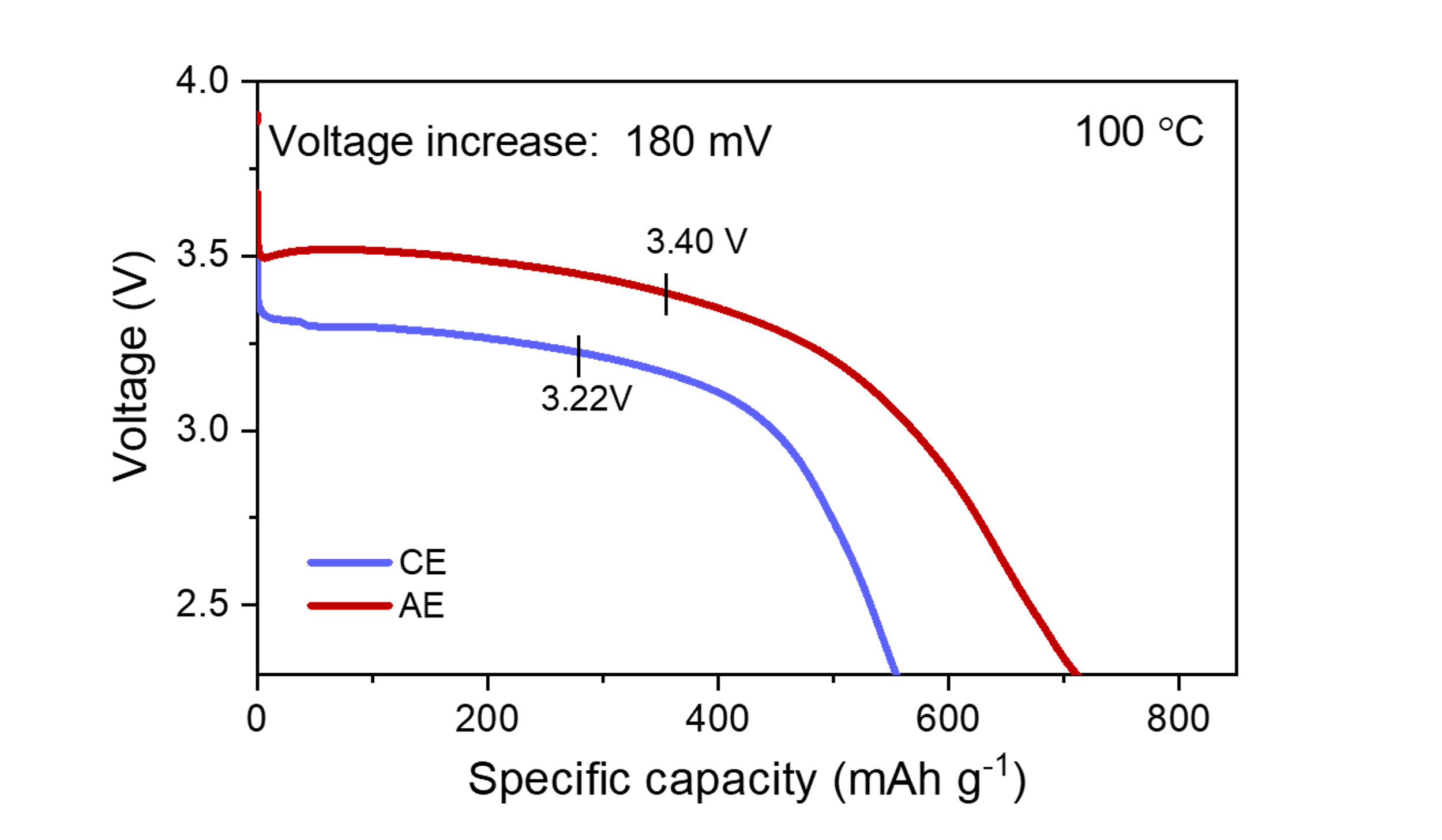


**Figure S11.** The discharge voltage profiles of the Li||CF*_x_* battery using AE and CE as electrolyte, respectively, measured at 100 ºC with a current density of 10 mA g^−1^.


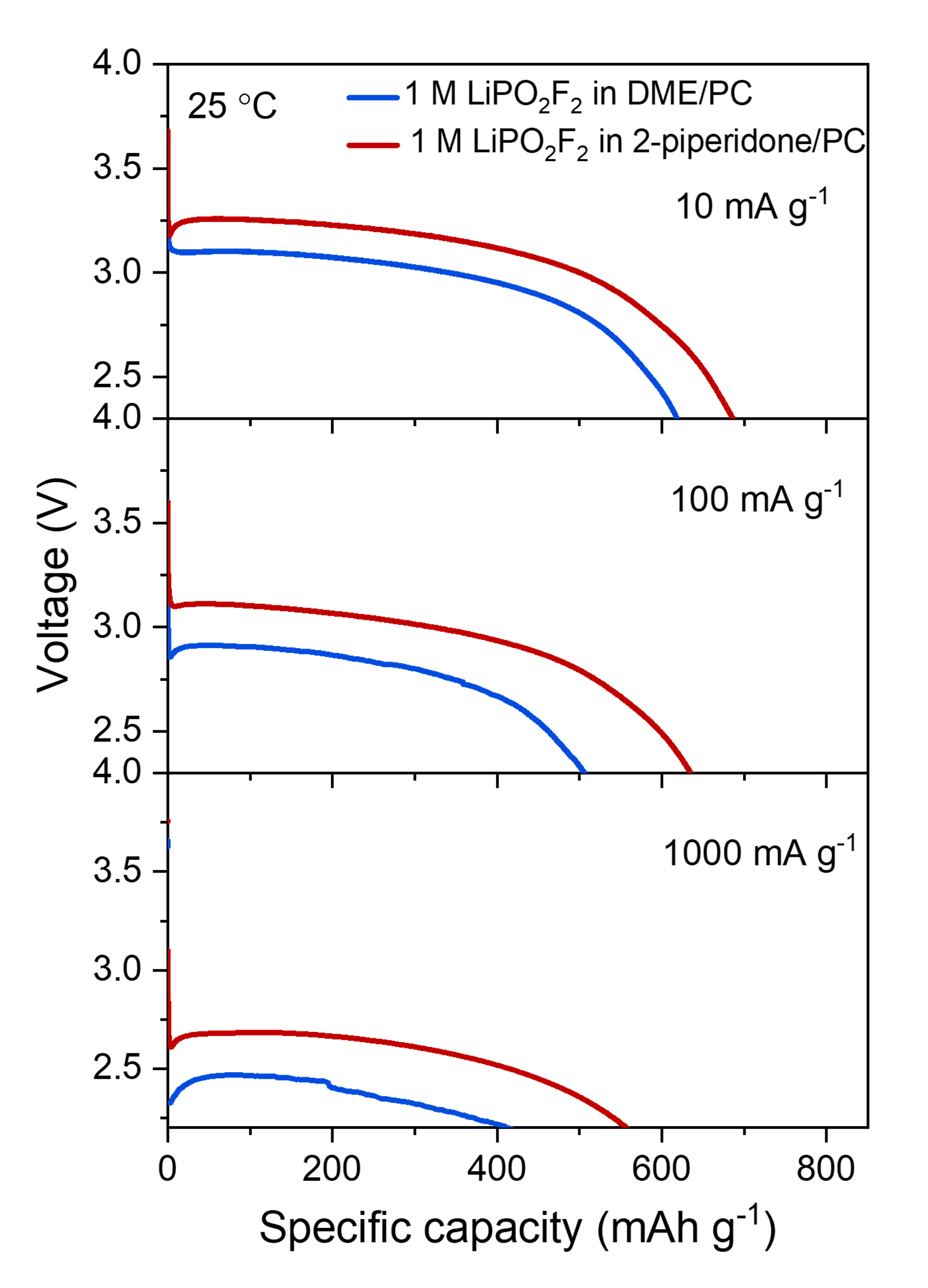


**Figure S12.** Rate performance of Li||CF*_x_* batteries using AE and CE as electrolyte, respectively.


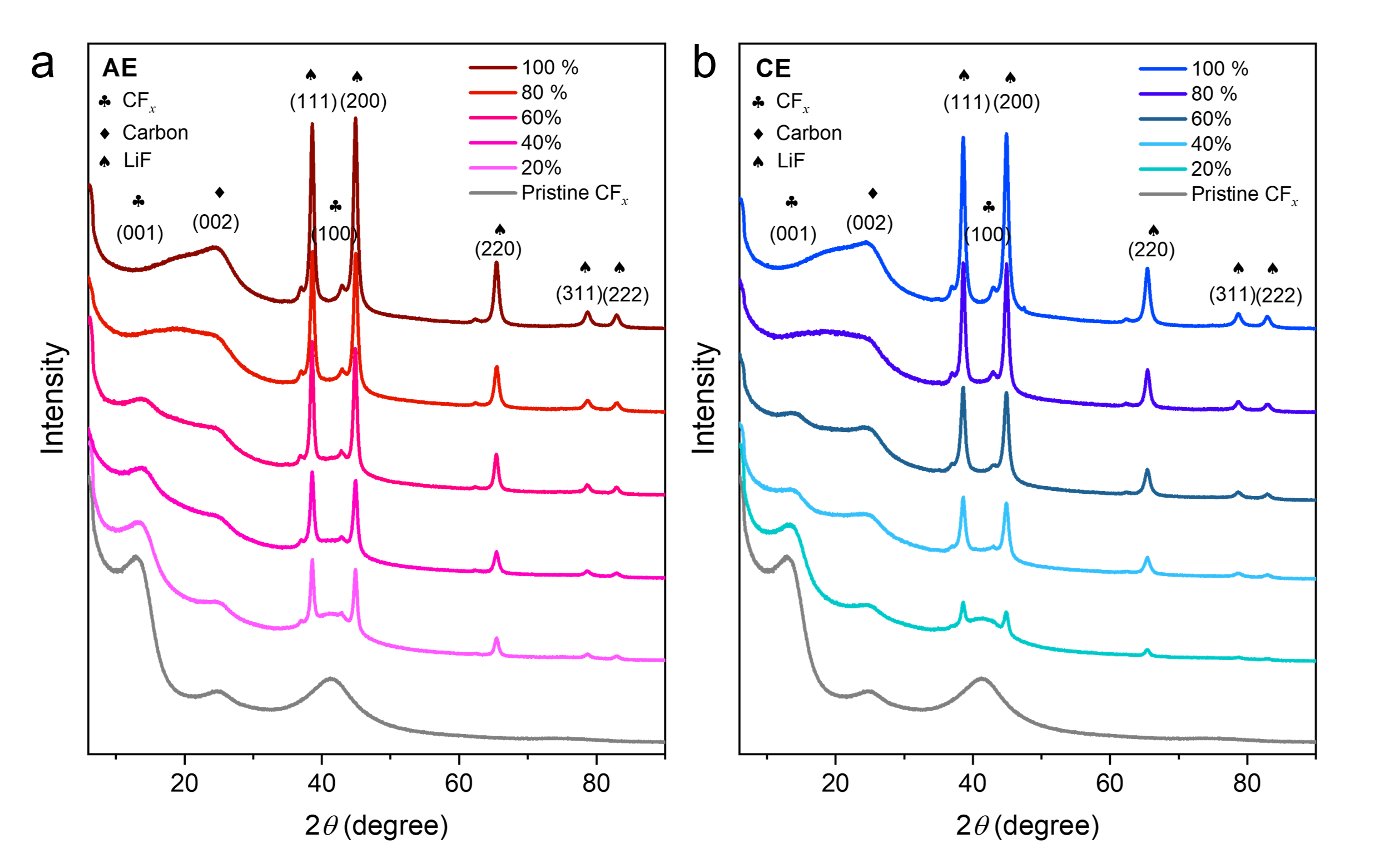


**Figure S13.** Ex situ XRD results of the CF*_x_* cathode at different depths of discharge, obtained from (a) AE and (b) CE electrolyte.

**
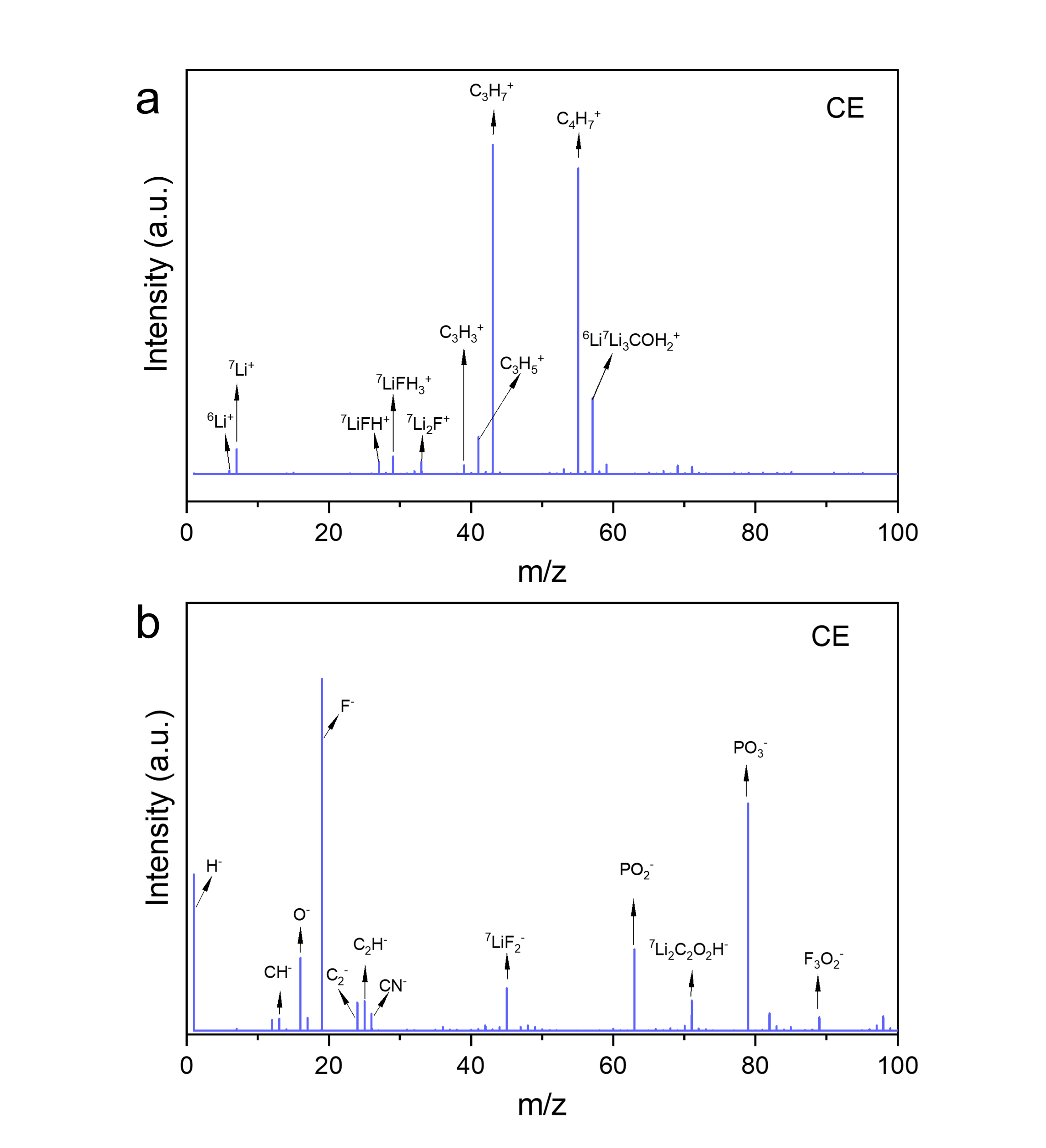
**

**Figure S14.** The TOF-SIMS spectrum of the CF*_x_* cathode discharged in CE. To avoid the distraction from PVDF, the samples are prepared without a binder.


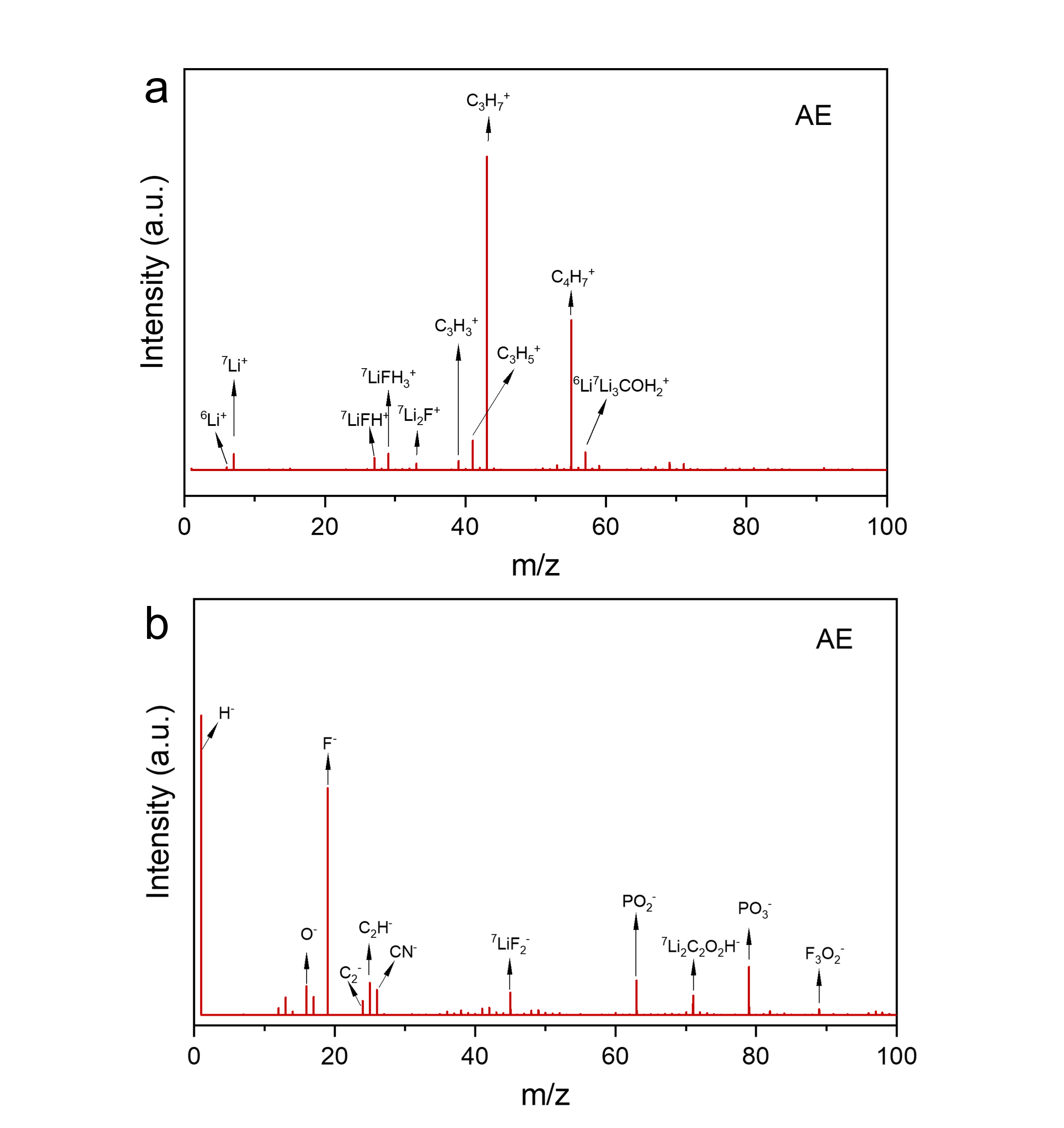


**Figure S15.** The TOF-SIMS spectrum of the CF*_x_* cathode discharged in AE. To avoid the distraction from PVDF, the samples are prepared without a binder.


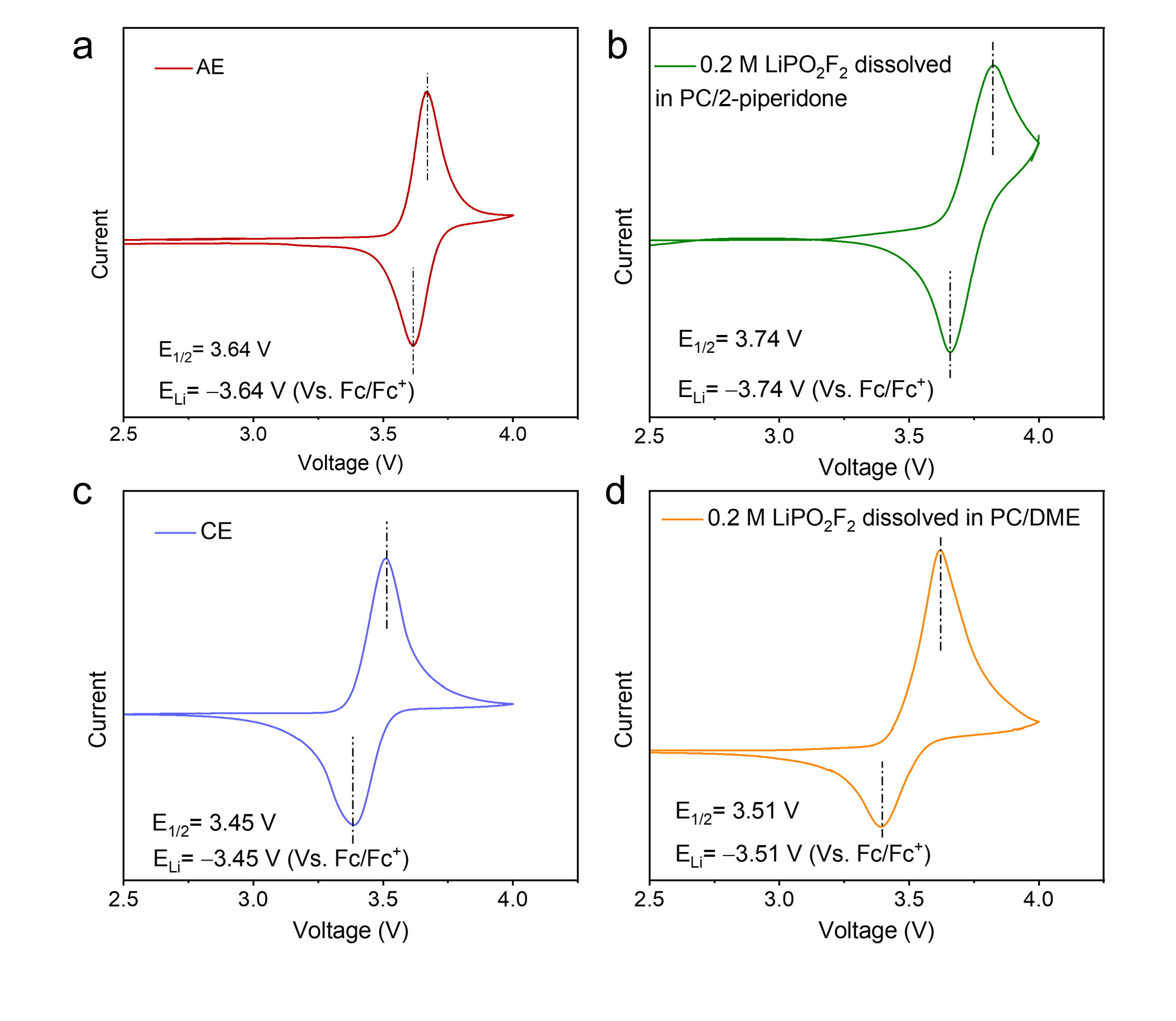


**Figure S16.** Lithium electrode potential calibrated with ferrocene in four electrolytes: (a) AE; (b) dilute AE; (c) CE, and (d) dilute CE.


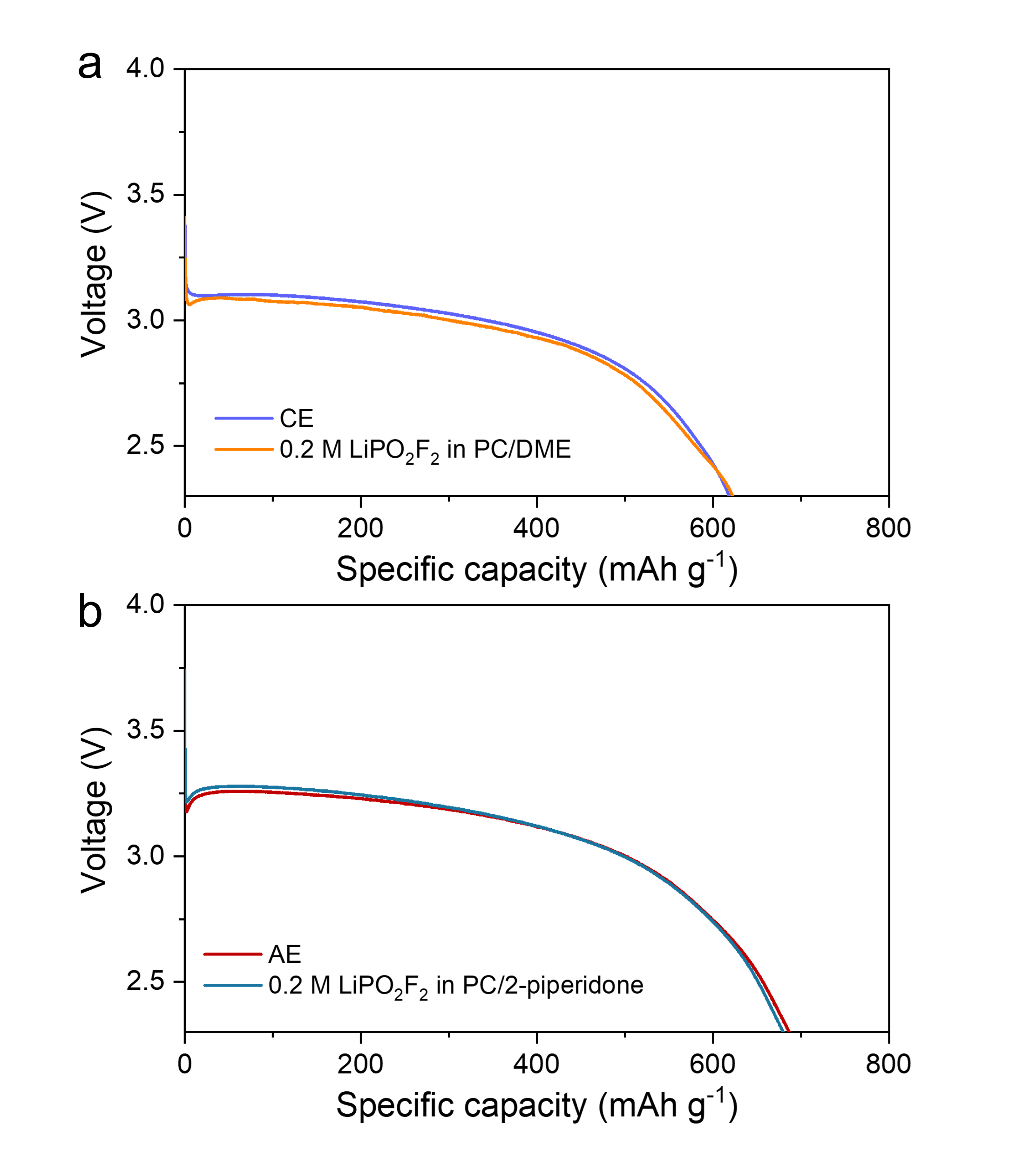


**Figure S17.** The discharge voltage comparison of the Li||CF*_x_* battery using (a) CE/dilute CE, and (b) AE/dilute AE. The batteries are measured at 25 ºC with a current density of 10 mA g^−1^.


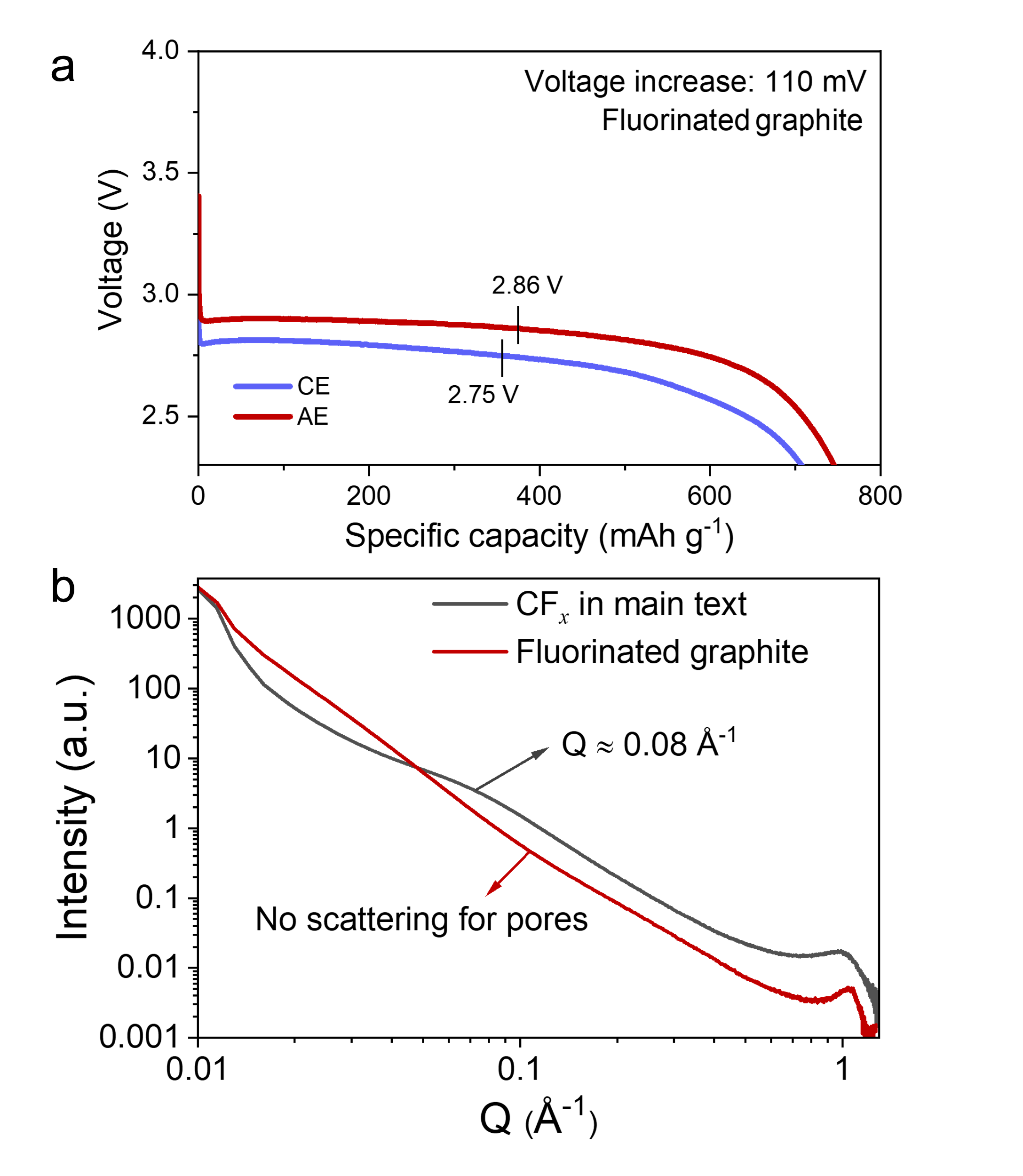


**Figure S18.** (a) Solvent-induced voltage enhancement phenomenon in fluorinated graphite. (b) SAXS profile of fluorinated graphite (red line): no scattering signal of pores is found. The SAXS data of CF*_x_* used in the main text is provided for comparison (grey line).


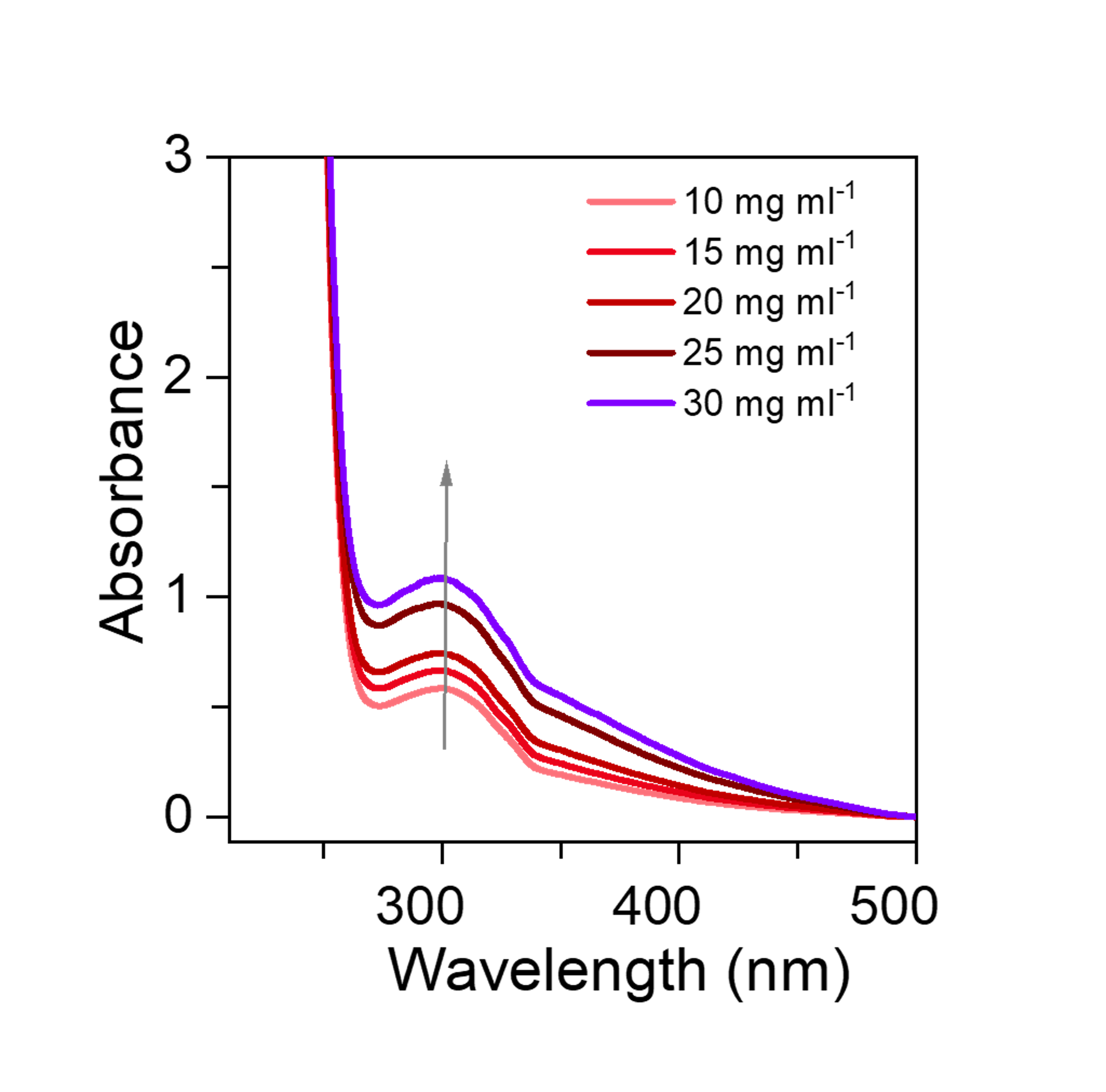


**Figure S19**. The electronic absorption spectra of CFx suspension using 2-piperidone/PC as solvents. The concentration of CFx suspension varies from 10 mg ml−1 to 30 mg ml−1. The spectra were baseline-corrected, and the absorbance at 500 nm was set to zero for normalization.


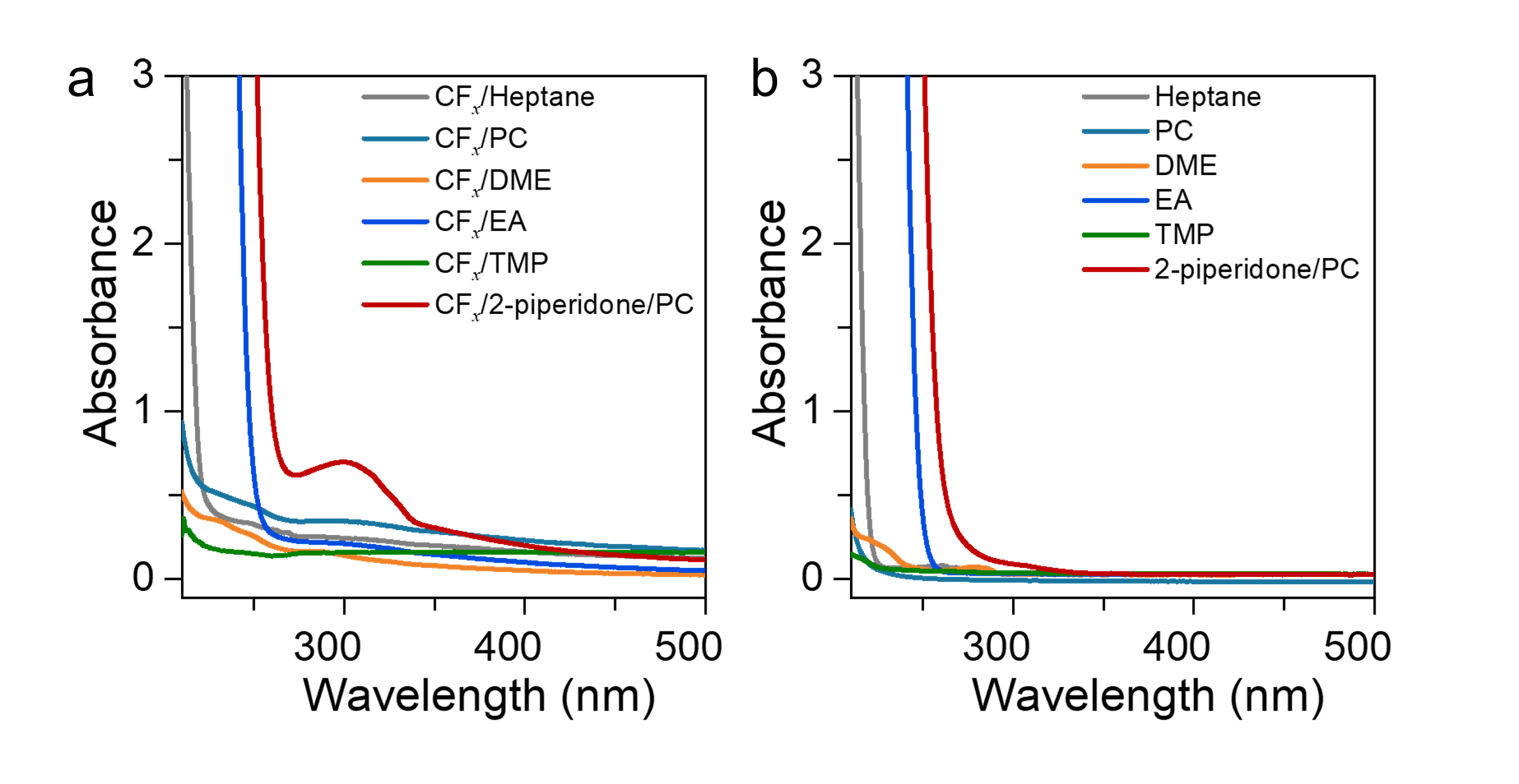
**Figure S20**. The electronic absorption spectra of CF_x_ suspension using heptane, PC, DME, EA, TMP, and 2-piperidone/PC as solvents, respectively, and their corresponding pure solvents.


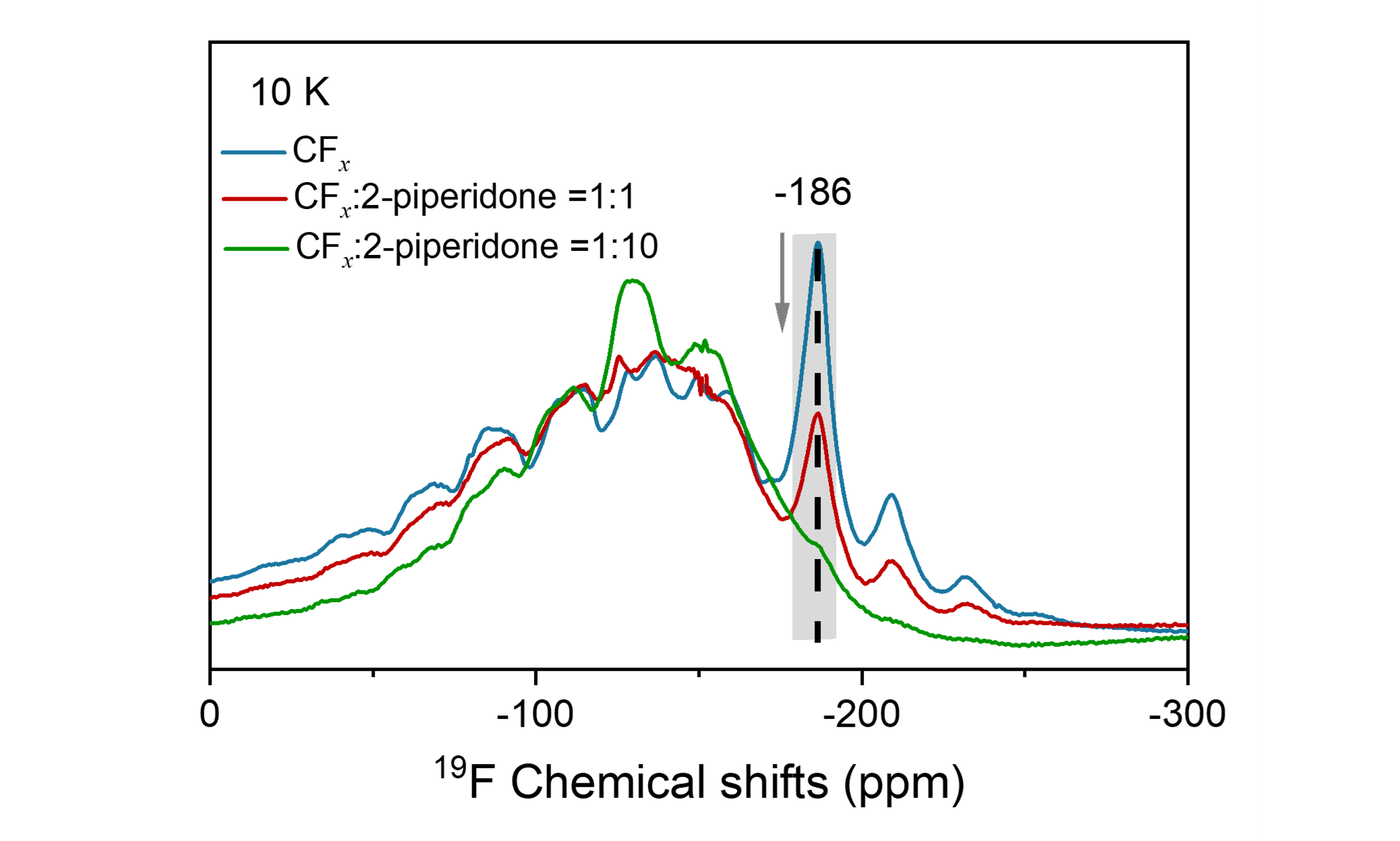


**Figure S21.** ^19^F solid-state NMR spectra of CF*_x_*, CF*_x_*/2-piperidone mixture (1:1 by weight), and CF*_x_*/2-piperidone mixture (1:10 by weight), spinning at 10 kHz.

Since the melting point of 2-piperidone is around 40ºC, to prevent liquefaction and vaporization of 2-piperidone during measurements, samples with higher 2-piperidone loading were acquired at a reduced spinning rate of 10 kHz. Low MAS frequencies reduce the experiment sensitivity and compromise the resolution of the ^19^F chemical shift. The bulge observed in the spectral curves originates from the interference of the rotor signal.


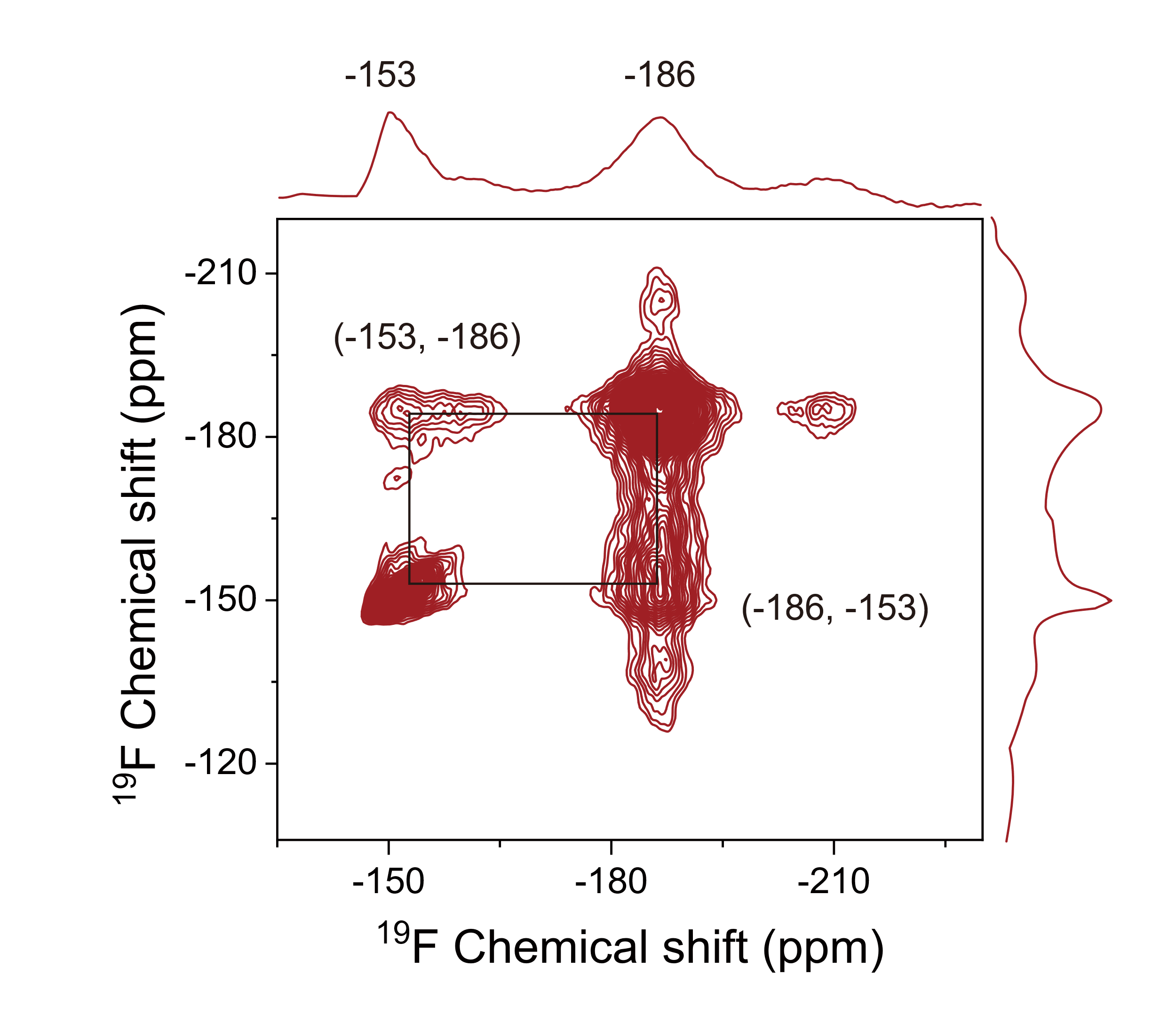


**Figure S22.** Two-dimensional ^19^F-^19^F homonuclear exchange MAS solid-state NMR spectra of CF*_x_* adsorbed with 2-piperidone, with a recoupling period of *τ*=20 ms.


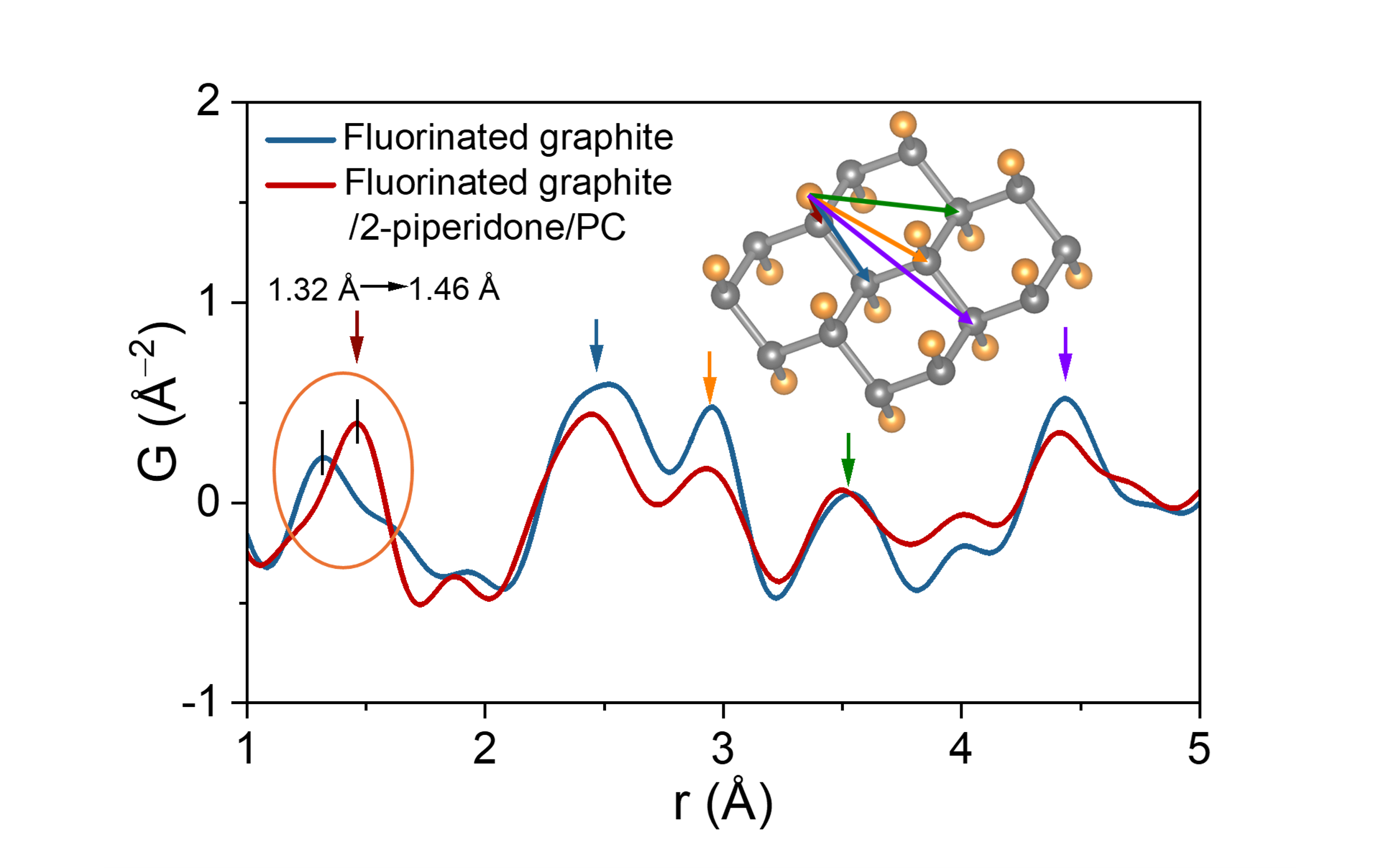


**Figure S23.** PDF analysis of fluorinated graphite with and without the solvent. The average C–F bond length extends from 1.32 Å to 1.46 Å.


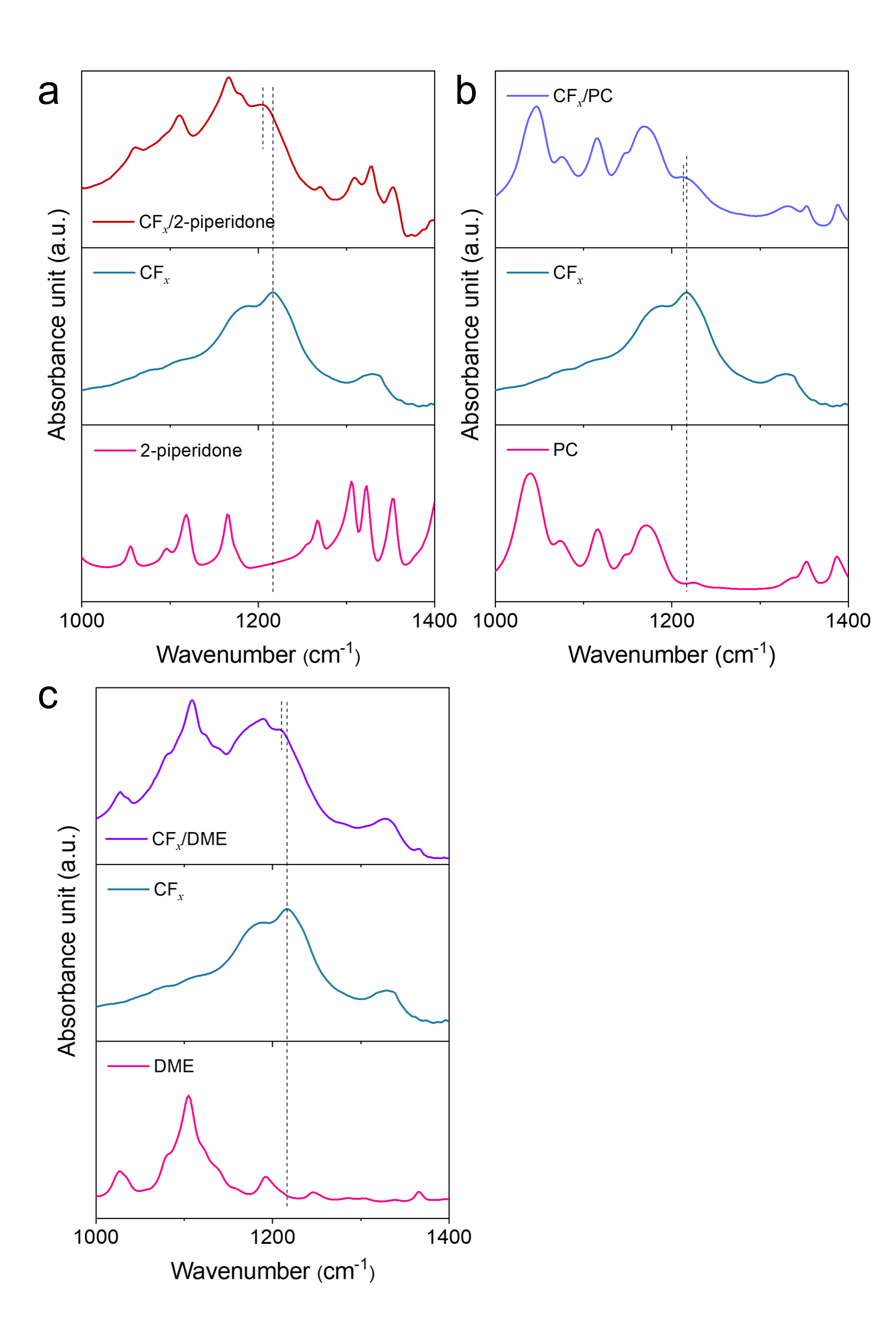


**Figure S24.** FTIR peak shift of CF*_x_* when immersed in (a) 2-piperidone, (b) PC and (c) DME.


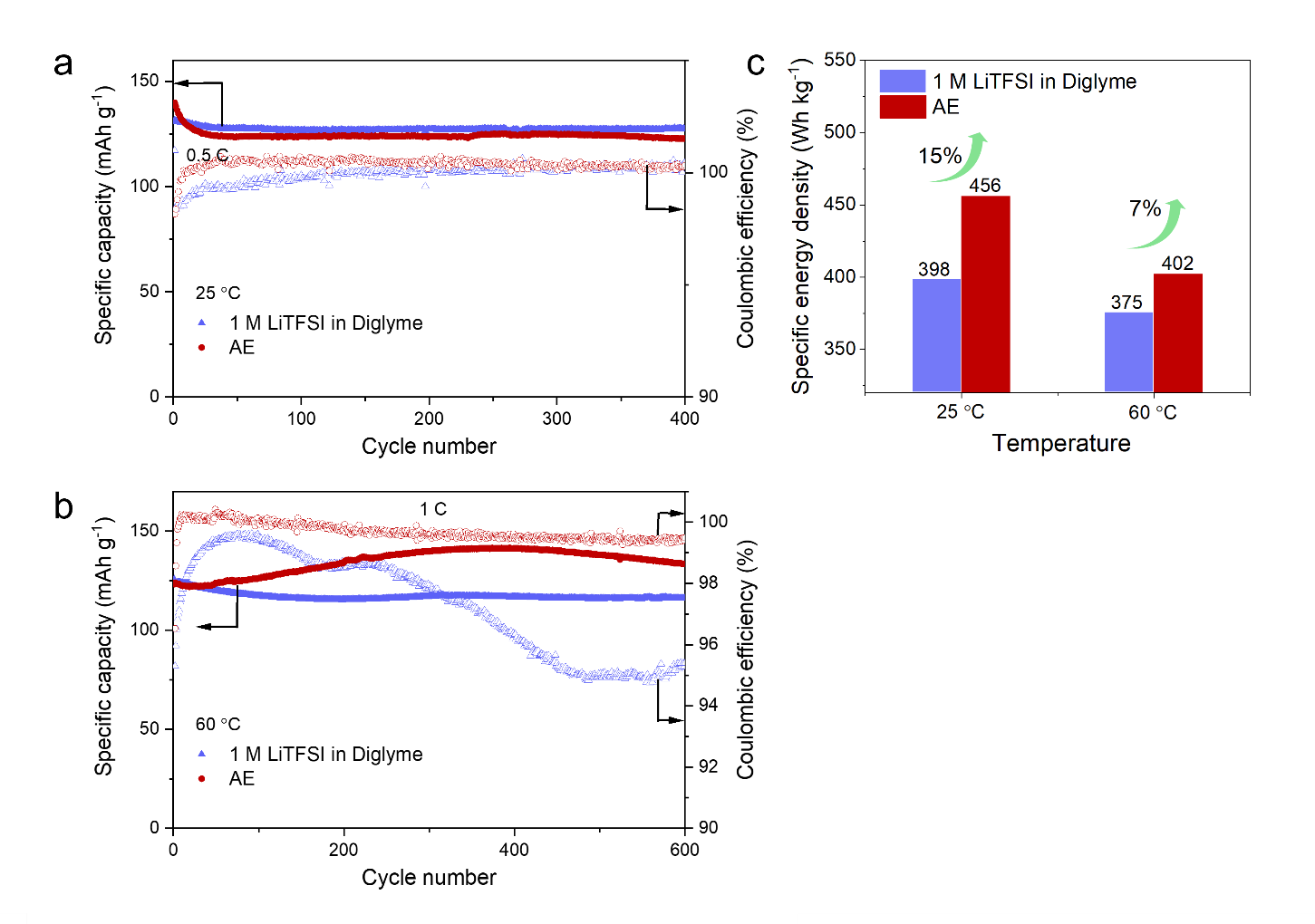


**Figure S25.** The cycling performance of Li||I_2_ batteries using AE and 1 M LiTFSI/diglyme as electrolyte, respectively, measured at (a) 25 ºC and (b) 60 ºC. AE exhibits excellent stability at high temperatures. (c) The energy density comparison based on the mass of the cathode.


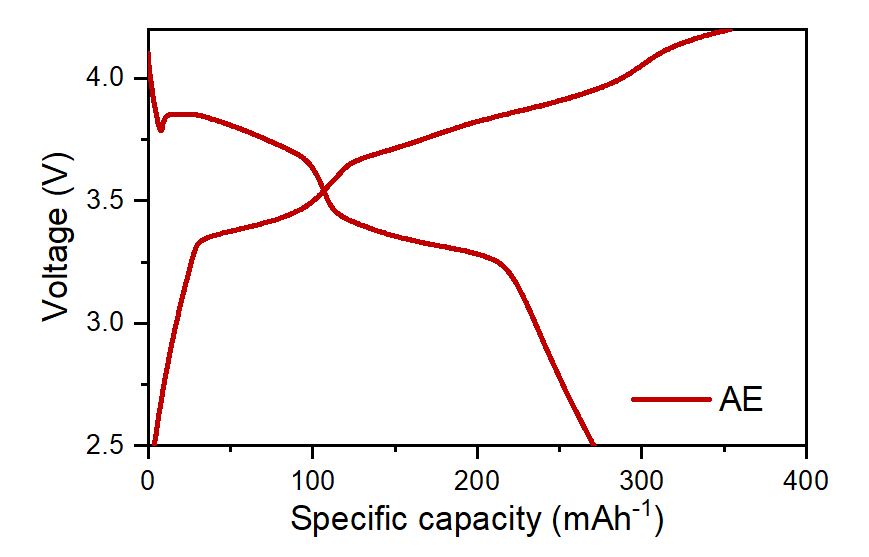


**Figure S26.** The charge/discharge profiles of the Li||I₂ battery within a voltage window of 2.5 V to 4.2 V.


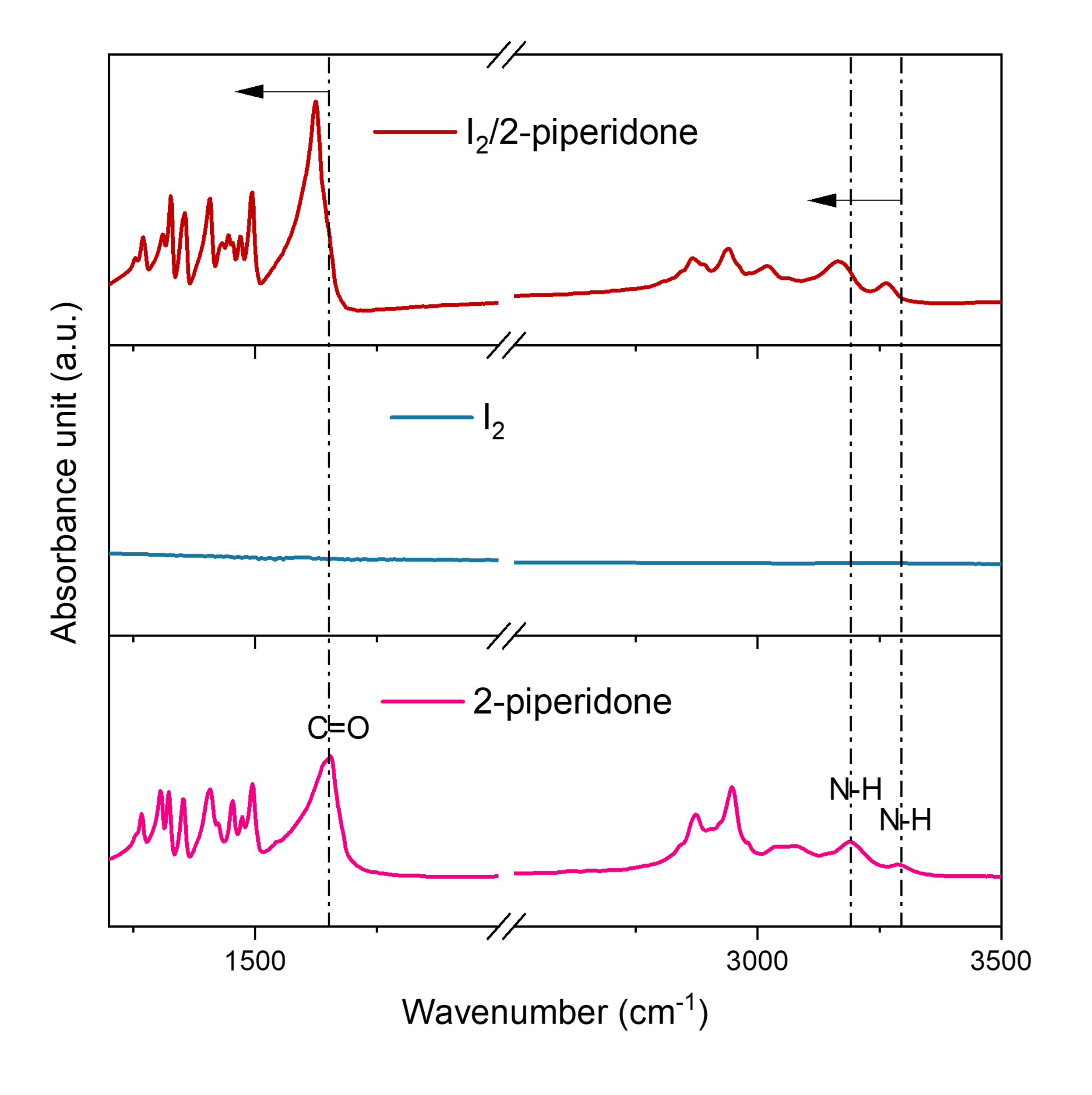


**Figure S27.** FTIR peak shift of 2-piperidone when I_2_ was dissolved in.


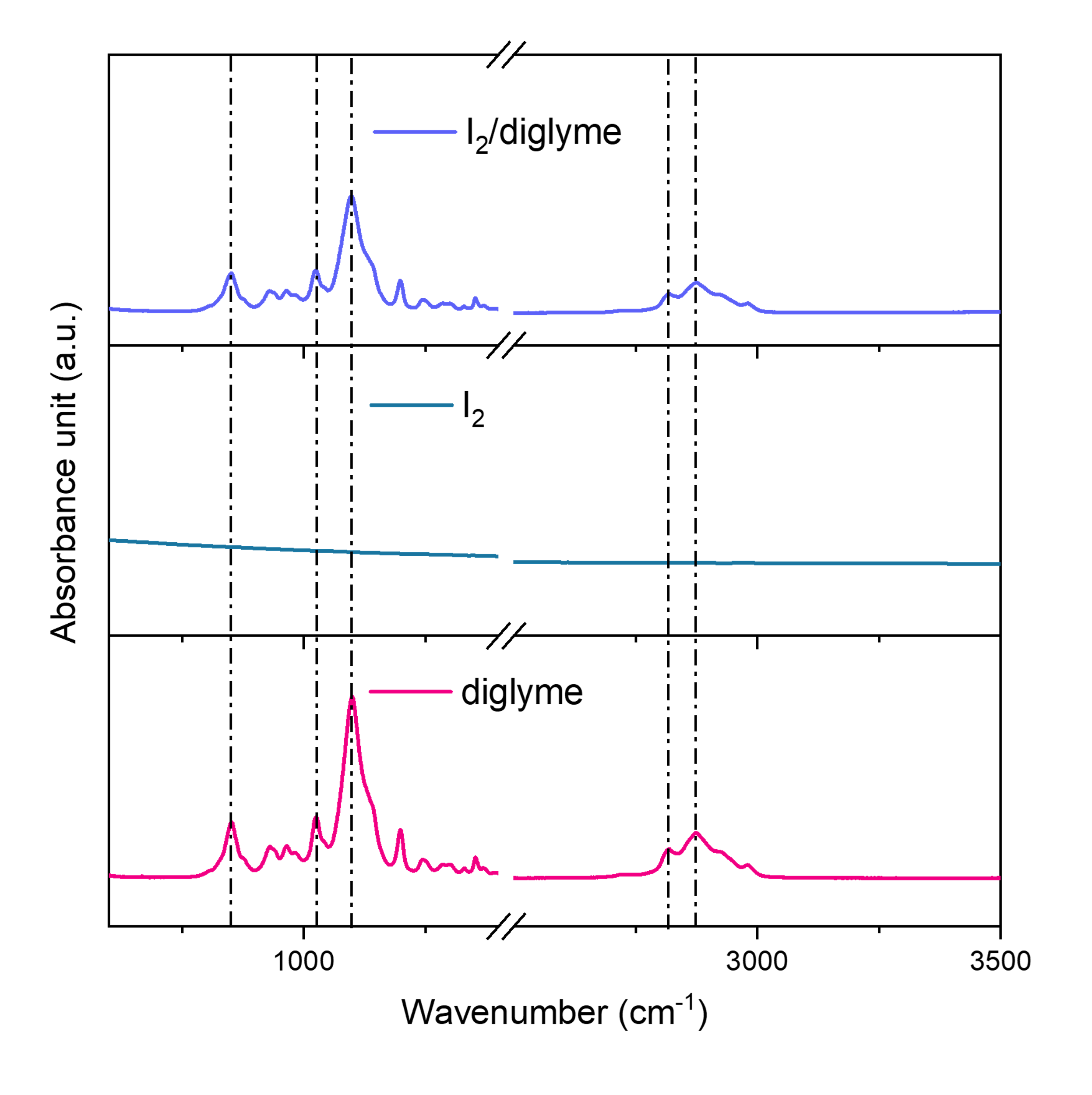


**Figure S28.** FTIR peak shift of diglyme when I_2_ was dissolved in.


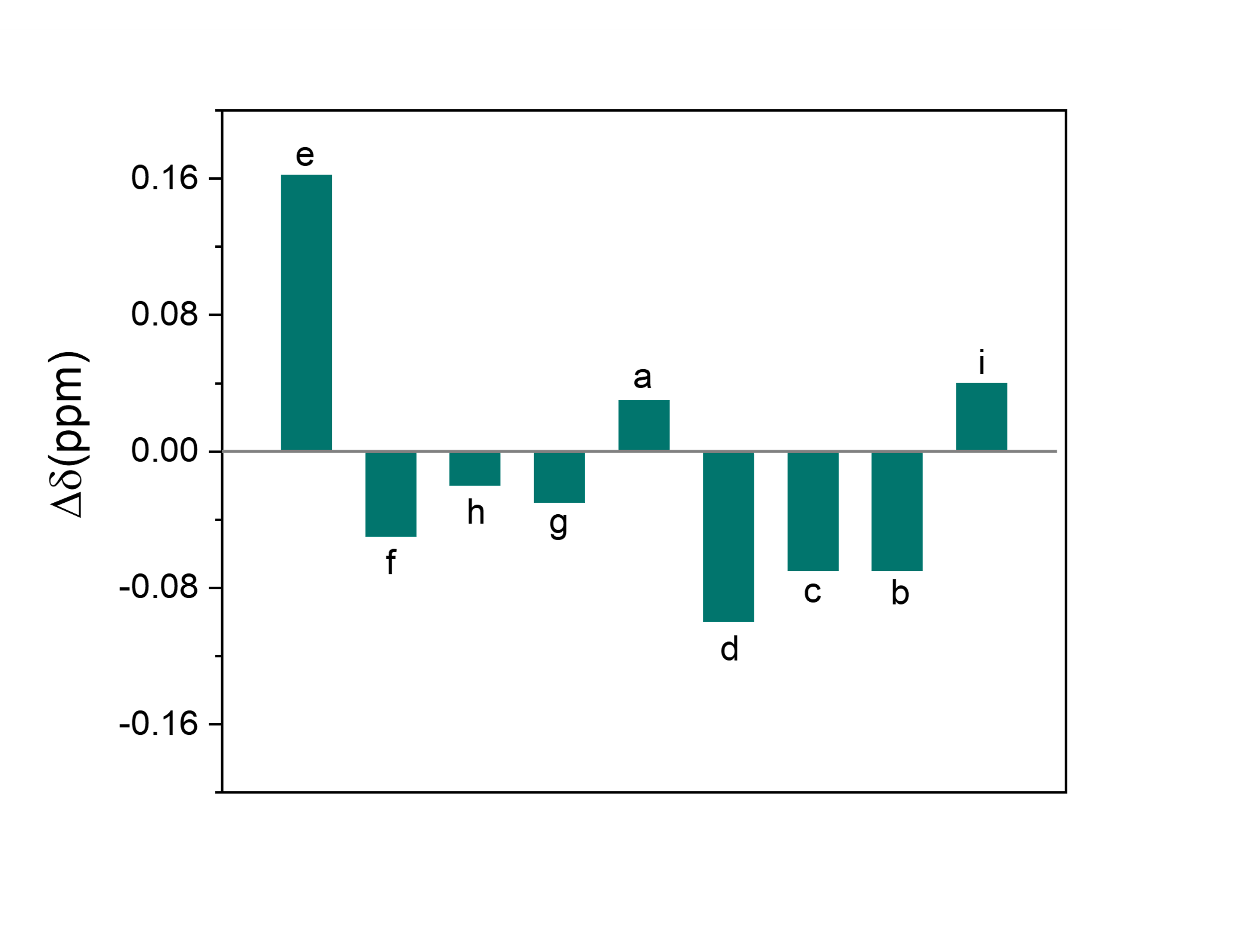


**Figure S29.** ^13^C NMR chemical shift changes of I_2_/2-piperidone/PC with respect to the 2-piperidone/PC solvent.


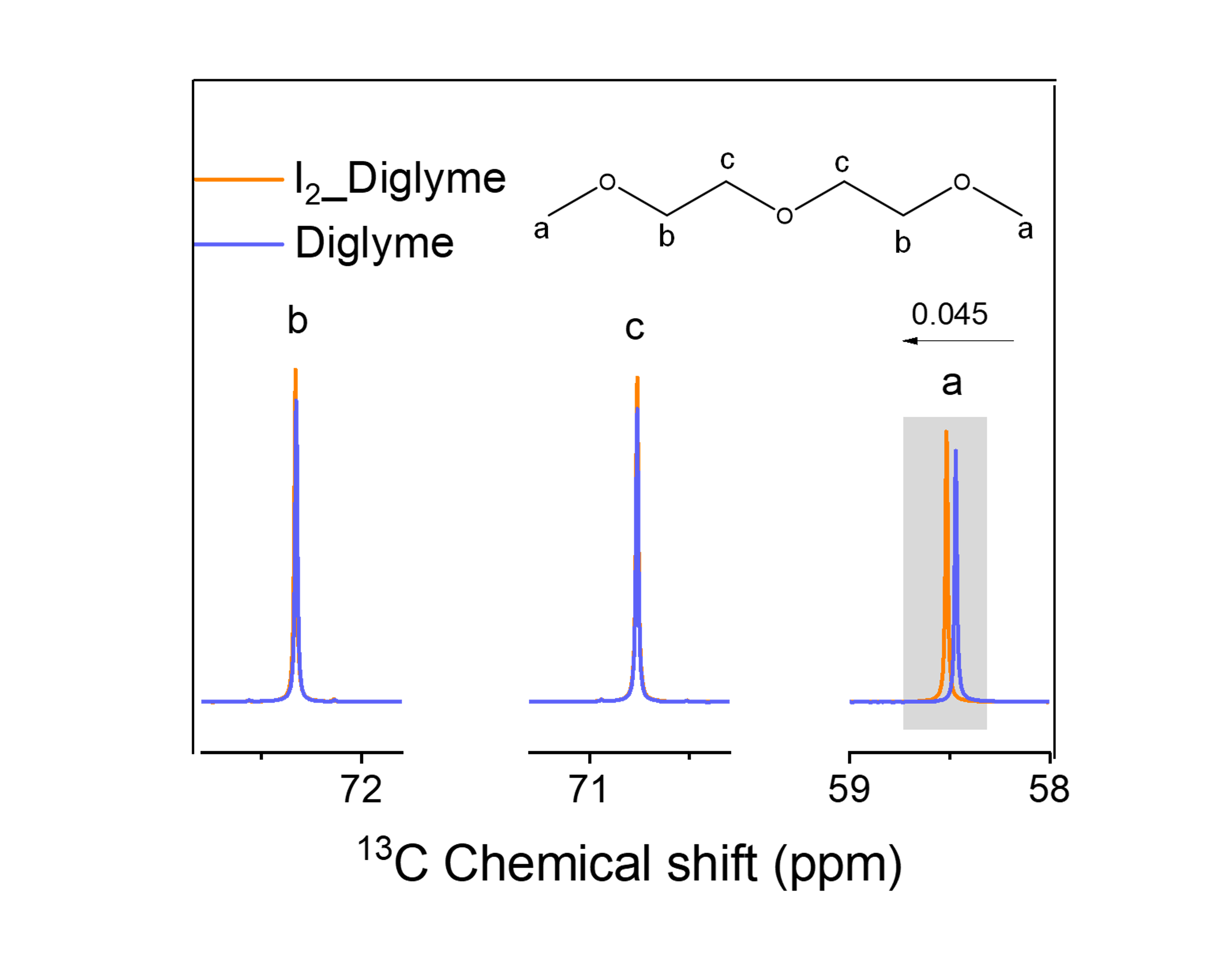


**Figure S30.** ^13^C NMR chemical shift of I_2_-diglyme and diglyme, showing a weak interaction.


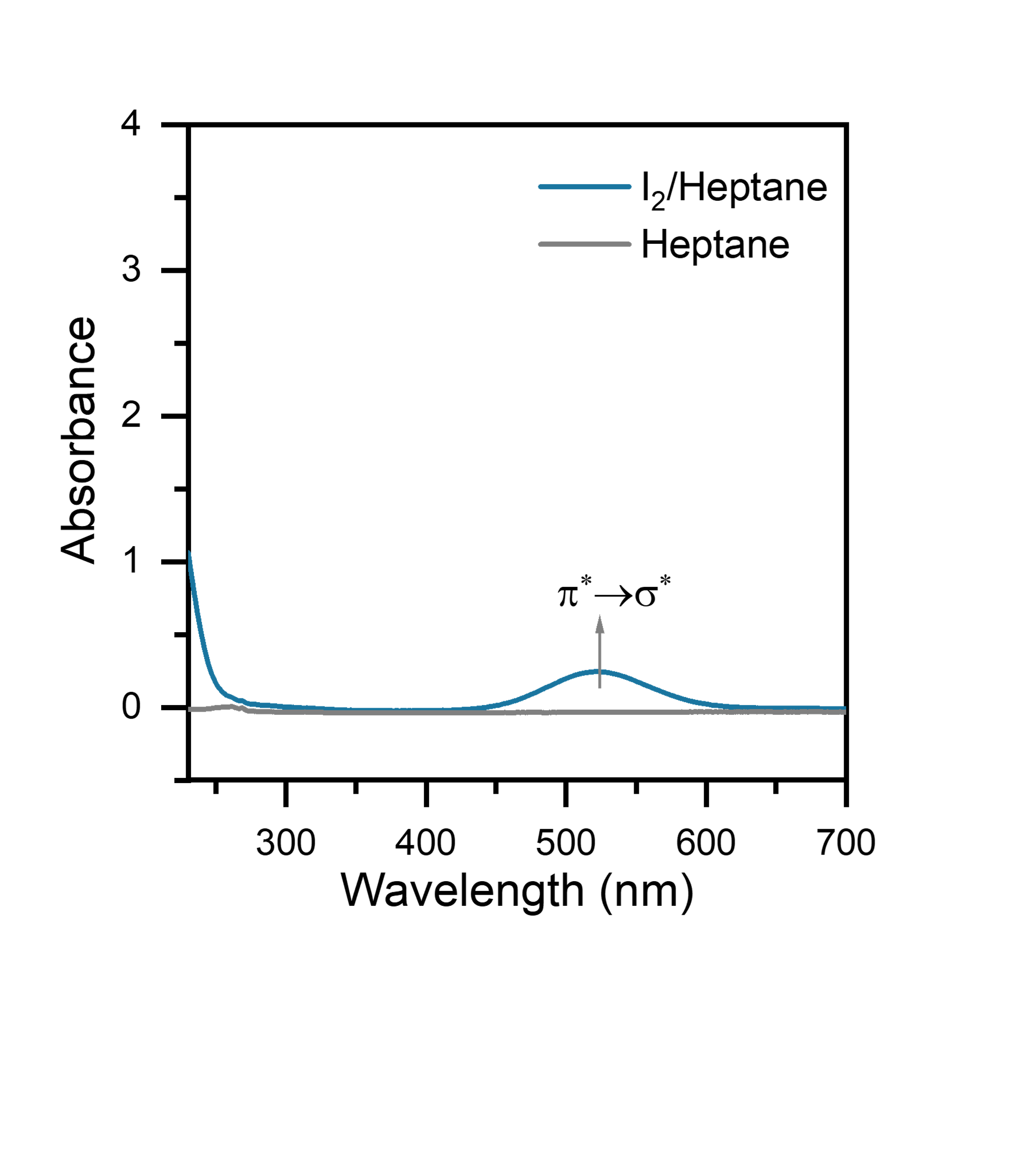


**Figure S31.** Electronic absorption spectra of 10 g L^−1^ I_2_/heptane solution and heptane.


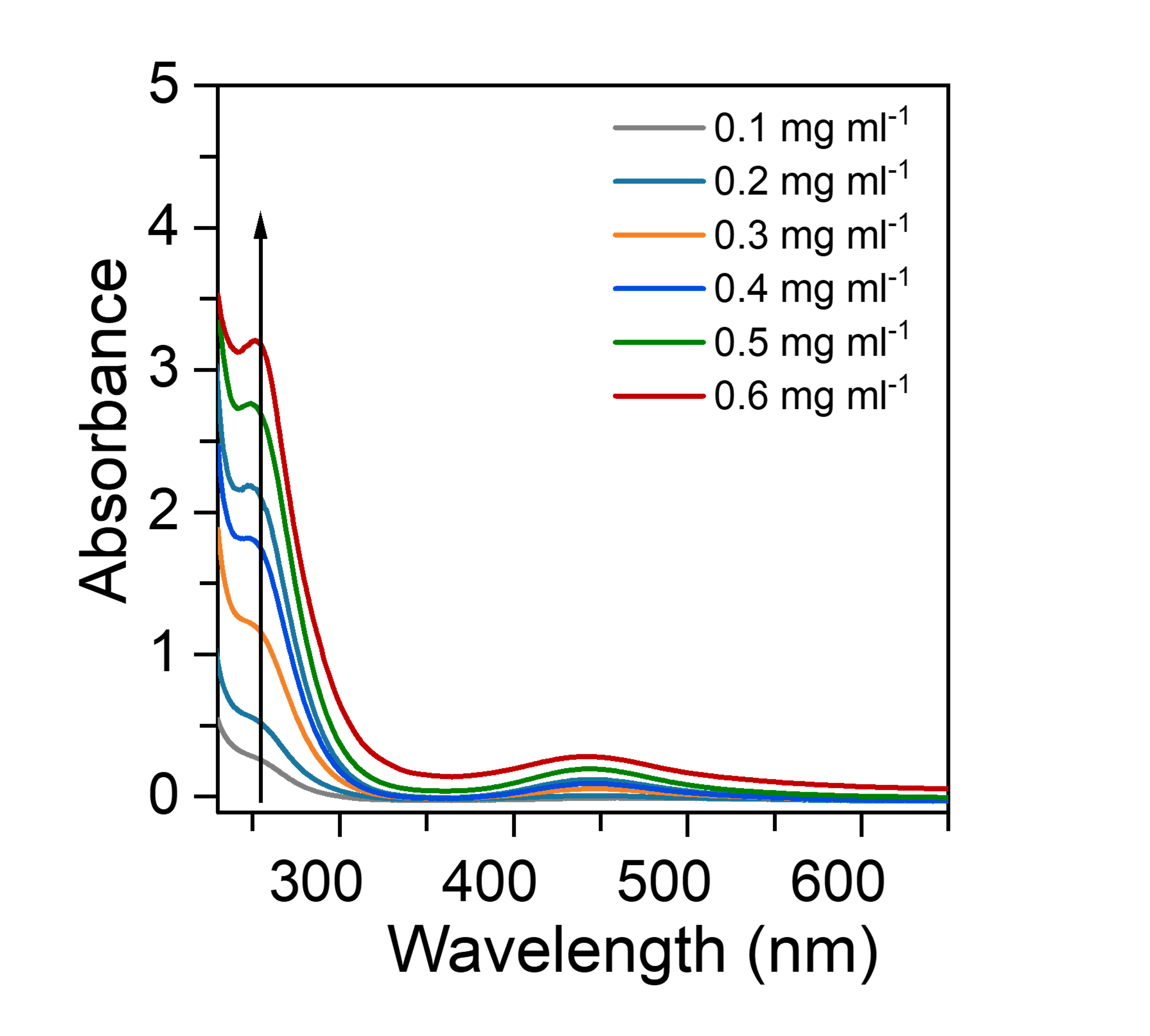


**Figure S32**. Electronic absorption spectra of I_2_ dissolved in 2-piperidone/heptane. The concentration of I_2_ varies from 0.1 mg ml^−1^ to 0.6 mg ml^−1^.


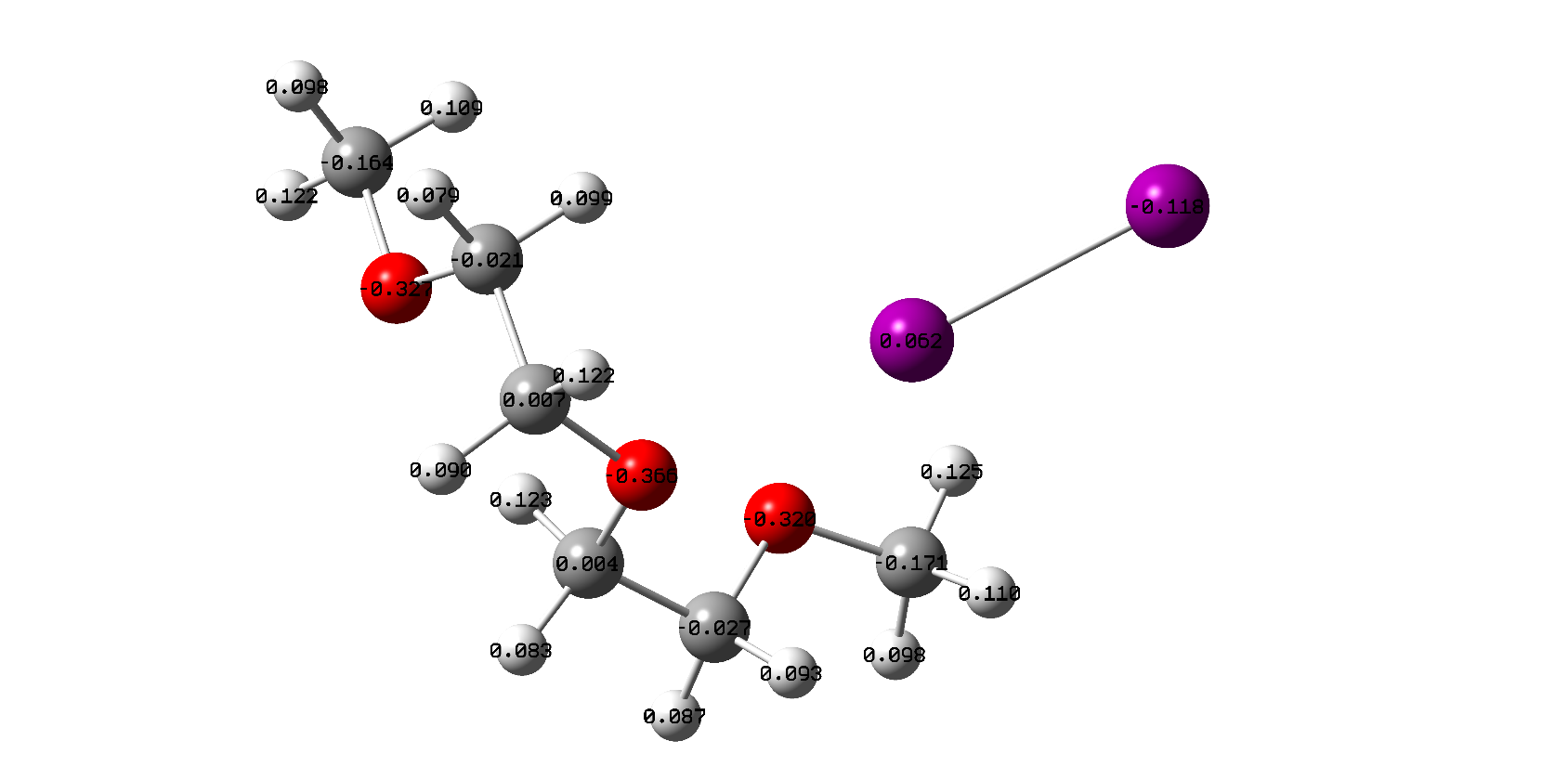


**Figure S33.** Charge population analysis of I_2_-diglymme. The value on the atom shows the charge change after interaction with I_2_.


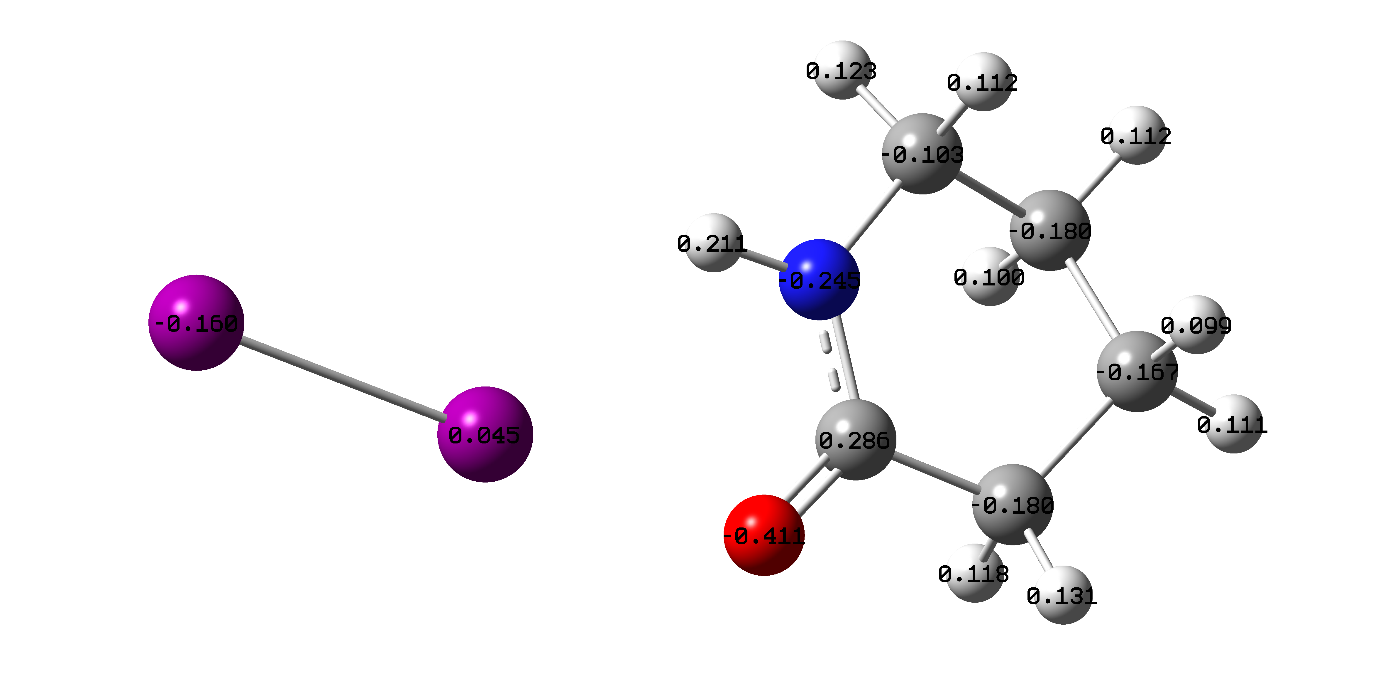


**Figure S34.** Charge population analysis of I_2_-2-piperidone. The value on the atom shows the charge change after interaction with I_2_.


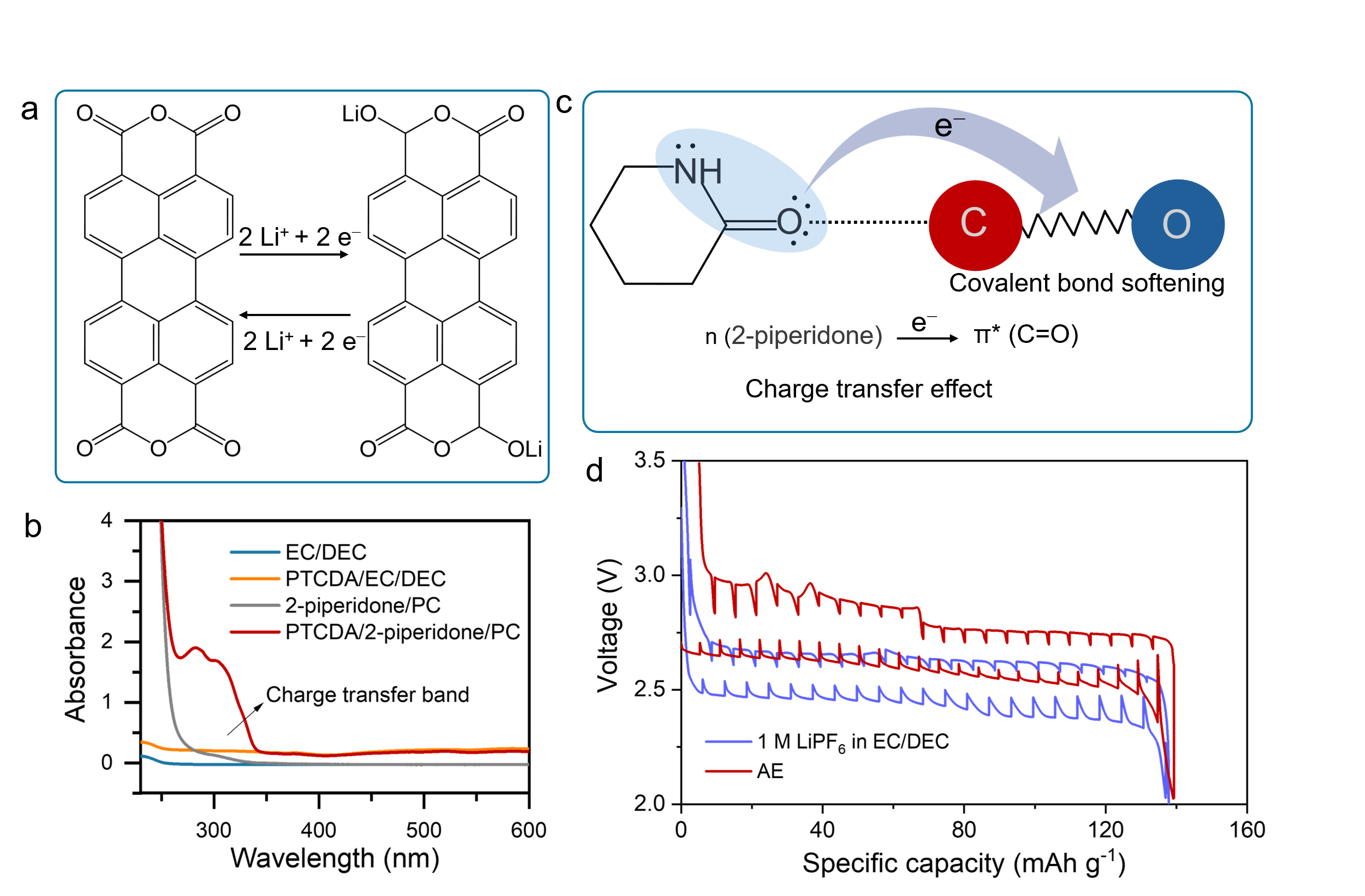


**Figure S35.** The application of the solvent-induced covalent bond softening strategy on Li||PTCDA battery. (a) The reversible reaction of Li||PTCDA battery; (b) The electronic absorption spectra of PTCDA suspension using EC/DEC and 2-piperidone/PC as solvent, respectively; (c) Charge transfer effect between 2-piperidone and PTCDA; (d) GITT test of Li||PTCDA batteries using the 1 M LiPF_6_ dissolved in EC/DEC and AE as electrolyte, respectively. Discussion refers to Supplementary Note 3.

**Reference**

[1] P. Juhás, T. Davis, C.L. Farrow, S.J.L. Billinge, PDFgetX3: a rapid and highly automatable program for processing powder diffraction data into total scattering pair distribution functions, Journal of Applied Crystallography 46(2) (2013) 560-566.

[2] A.D. Becke, Density-functional exchange-energy approximation with correct asymptotic behavior, Physical Review A 38(6) (1988) 3098-3100.

[3] C. Lee, W. Yang, R.G. Parr, Development of the Colle-Salvetti correlation-energy formula into a functional of the electron density, Physical Review B 37(2) (1988) 785-789.

[4] A.D. Becke, Density‐functional thermochemistry. III. The role of exact exchange, The Journal of Chemical Physics 98(7) (1993) 5648-5652.

[5] F. Weigend, R. Ahlrichs, Balanced basis sets of split valence, triple zeta valence and quadruple zeta valence quality for H to Rn: Design and assessment of accuracy, Physical Chemistry Chemical Physics 7(18) (2005) 3297-3305.

[6] W. Humphrey, A. Dalke, K. Schulten, VMD: Visual molecular dynamics, Journal of Molecular Graphics 14(1) (1996) 33-38.

[7] L. Johnson, C. Li, Z. Liu, Y. Chen, S.A. Freunberger, P.C. Ashok, B.B. Praveen, K. Dholakia, J.M. Tarascon, P.G. Bruce, The role of LiO_2_ solubility in O_2_ reduction in aprotic solvents and its consequences for Li-O_2_ batteries, Nat Chem 6(12) (2014) 1091-9.
